# Supplementary material for: Effect of muscle length on maximum evoked torque, discomfort, contraction fatigue, and strength adaptations during electrical stimulation in adult populations: A systematic review
Source: PLoS One. 2024 Jun 10;19(6):e0304205. doi: 10.1371/journal.pone.0304205 (PMC11164398; doi:10.1371/journal.pone.0304205)
Supplement: S2 File — (DOCX) [file pone.0304205.s002.docx]

**Supplemental Ditigal Content 2 –** Search Strategies according to Database

**PUBMED, Web of Science, BIREME, and Scielo**

(ALL=healthy OR ALL=adults OR ALL=participants OR ALL=volunteers OR ALL=muscle weakness OR ALL=muscle atrophy OR ALL=cachexia OR ALL=elderly OR ALL=muscle diseases OR ALL=muscle paralysis OR ALL=parkinson OR ALL=neuromuscular disease OR ALL=stroke OR ALL=multiple sclerosis OR ALL=anterior cruciate ligament reconstruction OR ALL=COPD OR ALL=lung disease OR ALL=pulmonary disease OR ALL=cardiac disease OR ALL=obese OR ALL=vascular disease OR ALL=diabetes OR ALL=orthopedic patients OR ALL=Nephrologic patients) AND (ALL=electric stimulation OR ALL=functional electrical stimulation OR ALL=neuromuscular electrical stimulation) AND (ALL=muscle length OR ALL=joint angle OR ALL=joint position) AND (ALL=torque OR ALL=force OR ALL=fatigue OR ALL=discomfort OR All=pain)

**EMBASE**

(healthy OR participants OR 'volunteers'/exp OR volunteers OR young OR 'adults'/exp OR adults) AND ('electric stimulation'/exp OR 'electric stimulation' OR 'functional electrical stimulation'/exp OR 'functional electrical stimulation' OR 'neuromuscular electrical stimulation'/exp OR 'neuromuscular electrical stimulation') AND ('muscle length'/exp OR 'muscle length' OR 'joint angle'/exp OR 'joint angle' OR 'joint position') AND ('torque'/exp OR torque OR 'force'/exp OR force OR 'fatigue'/exp OR fatigue OR 'discomfort'/exp OR discomfort OR 'pain'/exp OR pain)

**PEDro**

On the ‘Abstract & Title field’: electric* length angle

Note: all pre-established fields of the PEDro database were left blank because it retrieved no studies or did not affected the final result. All terms were matched by the AND opperator.

**Cochrane**

(All Text) healthy OR adults OR participants OR volunteers OR muscle weakness OR muscle atrophy OR cachexia OR elderly OR muscle diseases OR muscle paralysis OR parkinson OR neuromuscular disease OR stroke OR multiple sclerosis OR anterior cruciate ligament reconstruction OR COPD OR Lung disease OR pulmonary disease OR cardiac disease OR obese OR vascular disease OR diabetes OR orthopedic patients OR Nephrologic patients AND (All Text) electric stimulation OR functional electrical stimulation OR neuromuscular electrical stimulation AND (All Text) muscle length OR joint angle OR joint position AND (All Text) torque OR force OR fatigue OR discomfort OR pain
