# Supplementary material for: Effect of muscle length on maximum evoked torque, discomfort, contraction fatigue, and strength adaptations during electrical stimulation in adult populations: A systematic review
Source: PLoS One. 2024 Jun 10;19(6):e0304205. doi: 10.1371/journal.pone.0304205 (PMC11164398; doi:10.1371/journal.pone.0304205)
Supplement: S3 File — (PDF) [file pone.0304205.s003.pdf]

Intention-to-treat

Unique ID	Study ID	Experimental	Comparator	Outcome	Weight	D1	DS	D2	D3	D4	D5	Overall
Cavalcante et al., 2022	Cavalcante et al., 2022	NA	NA	Fatigue	1	+	+	+	+	+	+	+
Cavalcante et al., 2021	Cavalcante et al., 2021	NA	NA	Torque	1	+	+	+	+	+	+	+
Harnie et al., 2020	Harnie et al., 2020	NA	NA	Peak twitch torque	1	+	+	+	+	+	+	+
Hali et al., 2020	Hali et al., 2020	NA	NA	Peak twitch torque	1	+	+	+	+	+	+	+
Fouré et al., 2019	Fouré et al., 2019	NA	NA	Muscle soreness	1	+	!	+	+	+	+	!
Debenham & Power, 2019	Debenham & Power, 2019	NA	NA	Rate of Torque Develop	1	!	!	+	+	!	+	-
Scott et al., 2019	Scott et al., 2019	NA	NA	Evoked Torque	1	+	+	+	+	+	+	+
Gavin et al., 2018	Gavin et al., 2018	NA	NA	Discomfort	1	+	-	+	+	+	+	-
Merlet et al., 2018	Merlet et al., 2018	NA	NA	Twitch torque	1	+	+	+	+	+	+	+
Yanase et al., 2017	Yanase et al., 2017	NA	NA	Muscle swelling and soı	1	+	+	+	+	+	+	+
Visscher et al., 2017	Visscher et al., 2017	NA	NA	Peak twitch torque	1	+	+	+	+	+	+	+
Bampouras et al., 2017	Bampouras et al., 2017	NA	NA	Tetanic Torque	1	+	!	+	+	+	+	!
Ando et al., 2016	Ando et al., 2016	NA	NA	Contraction Fatigability	1	+	+	+	+	+	+	+
Bremner et al., 2015a	Bremner et al., 2015a	NA	NA	Evoked torque	1	+	+	+	+	+	+	+
Bremner et al., 2015b	Bremner et al., 2015b	NA	NA	Perceived discomfort	1	+	+	+	+	+	+	+
Frigon et al., 2011	Frigon et al., 2011	NA	NA	Extra forces (MIscelane	1	+	+	+	+	+	+	+
Skurvydas et al., 2010	Skurvydas et al., 2010	NA	NA	Evoked torque	1	+	!	+	+	+	+	!
Marion et al., 2009	Marion et al., 2009	NA	NA	Fatigue	1	+	+	+	+	+	+	+
Lee et al., 2007	Lee et al., 2007	NA	NA	Fatigue	1	+	+	+	+	+	+	+
Guerrits et al., 2005	Guerrits et al., 2005	NA	NA	Evoked torque through	1	+	+	+	+	+	+	+
Miyamoto & Oda, 2005	Miyamoto & Oda, 2005	NA	NA	30 Hz Tetanic torque; P	1	+	!	+	+	+	+	!
Kooistra et al., 2004	Kooistra et al., 2005	NA	NA	Triplet Torque	1	+	+	+	+	+	+	+
Maffiuletti et al., 2003	Maffiuletti et al., 2003	NA	NA	Peak twitch torque	1	+	+	+	+	+	+	-
Hansen et al., 2003	Hansen et al., 2003	NA	NA	Single and Doublet Twi	1	+	!	+	+	+	+	!
Mela et al., 2001	Mela et al., 2001	NA	NA	Peak Twitch torque	1	+	!	+	+	+	+	!
Rassier, 2000	Rassier, 2000	NA	NA	Twitch torque; Fatigue	1	+	+	+	+	+	+	+
Sacco et al., 1994	Sacco et al., 1994	NA	NA	Fatigue	1	-	+	+	+	+	+	-
McNeal & Bake, 1988	McNeal & Bake, 1988	NA	NA	Evoked Toruqe	1	-	+	+	+	+	+	-
Fitch & McComas, 1985	Fitch & McComas, 1985	NA	NA	Fatigue	1	+	+	+	+	+	+	+
Fahey et al., 1984	Fahey et al., 1984	NA	NA	Strengthening for isom	1	+	+	+	+	+	+	+
Marsh et al., 1981	Marsh et al., 1981	NA	NA	Tetanic stimulation at 4	1	+	-	+	+	+	+	-
Kooistra et al., 2007	Kooistra et al., 2007	NA	NA	NA	1	+	+	+	+	+	+	+
Ruiter et al., 2004	Ruiter et al., 2004	NA	NA	NA	1	+	+	+	+	+	+	+

\+ Low risk
! Some concerns
\- High risk

D1	Randomisation process
DS	Bias arising from period and carryover effects
D2	Deviations from the intended interventions
D3	Missing outcome data
D4	Measurement of the outcome
D5	Selection of the reported result

Ruiter et al., 2008	Ruiter et al., 2008	NA	NA	Triplet torque	1	+	+	+	+	+	+	+
Place et al., 2004	Place et al., 2004	NA	NA	Peak twitch torque	1	+	+	+	+	+	+	+
Babault et al., 2003	Babault et al., 2003	NA	NA	Peak twitch torque	1	+	+	+	+	+	+	+
