# Supplementary material for: Effect of muscle length on maximum evoked torque, discomfort, contraction fatigue, and strength adaptations during electrical stimulation in adult populations: A systematic review
Source: PLoS One. 2024 Jun 10;19(6):e0304205. doi: 10.1371/journal.pone.0304205 (PMC11164398; doi:10.1371/journal.pone.0304205)
Supplement: Attachment — Submitted filename: Manuscript-numbered.docx [file pone.0304205.s004.docx]

**Effect of muscle length on maximum evoked torque, discomfort, fatigue, and strength adaptations during electrical stimulation in adult populations: a systematic review**

**Authors:**

Jonathan Galvão Tenório Cavalcante, Msc^a^, Victor Hugo de Souza Ribeiro, PT^b^, Rita de Cássia Marqueti, PhD^c^, Isabel de Almeida Paz, Msc^d^, Júlia Aguillar Ivo Bastos, Msc^b^, Marco Aurélio Vaz, PhD^d^, Nicolas Babault, PhD^e^, João Luiz Quaglioti Durigan, PhD^a,b^

**Affiliations:**

^a^ University of Brasília, Laboratory of Muscle and Tendon Plasticity, Graduate Program of Physical Education, Distrito Federal, Brazil.

^b^ University of Brasília, Laboratory of Muscle and Tendon Plasticity, Graduate Program of Rehabilitation Sciences, Distrito Federal, Brazil.

^c^ University of Brasília, Molecular Analysis Laboratory, Graduate Program of Rehabilitation Sciences, Distrito Federal, Brazil.

^d^ Exercise Research Laboratory - School of Physical Education, Physical Therapy and Dance - Federal University of Rio Grande do Sul - Porto Alegre, RS, Brazil.

^e^ Centre d’Expertise de la Performance, INSERM UMR1093-CAPS, University of Burgundy Franche-Comté, UFR des Sciences du Sport, F-21000, Dijon.

**Correspondence:**

João Luiz Quagliotti Durigan, PT, MS, PhD

Laboratory of Muscle and Tendon Plasticity

Graduate Program in Rehabilitation Sciences

University of Brasília,

Ceilandia, DF, Brasil, CEP 72220-140,

E-mail:durigan@unb.br

**Abstract**

Neuromuscular electrical stimulation (NMES) can improve physical function in different populations. NMES-related outcomes may be influenced by muscle length (i.e., joint angle), a modulator of the force generation capacity of muscle fibers. Nevertheless, a comprehensive synthesis of scientific evidence regarding the optimal joint angle for maximizing the effectiveness of NMES has yet to be provided. We aimed to investigate the effect of muscle length on NMES-induced isometric torque, discomfort, fatigue, and strength training adaptations in healthy and clinical adult populations through a systematic review (PROSPERO: CRD42022332965). We conducted searches across seven electronic databases: PUBMED, Web Of Science, EMBASE, PEDro, BIREME, SCIELO, and Cochrane, covering the period from June 2022 to October 2023, without restricting the publication year. We included cross-sectional and longitudinal studies that used NMES as an intervention or assessment tool for comparing muscle lengths in any adult population. We excluded studies on vocalization, respiratory, or pelvic floor muscles. Data extraction was performed via a standardized form to gather information on participants, interventions, and outcomes. The risk of bias was assessed using the Revised Cochrane risk-of-bias tool for cross-over trials (RoB-CO) and the Physiotherapy Evidence Database (PEDro) scale. Out of the 1185 articles retrieved through our search strategy, we included 36 studies in our analysis enrolling 448 healthy young participants (age: 19 - 40 years) to investigate maximum evoked torque (n=268), contraction fatigability (n=87), discomfort (n=82), and muscle strengthening (n=22), besides six participants with spinal cord injuries, and 15 healthy old participants. Meta-analyses were possible only for comparing maximal evoked torque according to quadriceps muscle length through knee joint angle. At optimal muscle length (55º - 65º of knee flexion, where 0º is full extension), there was greater evoked torque compared to very short (0 – 30º) (p<0.001, CI 95%: -2.03, -1.15 for muscle belly stimulation, and -3.64, -1.10 for femoral nerve stimulation) and long (66º - 90º) (p<0.001, CI 95%: 0.48, 1.22) muscle lengths. At long muscle lengths, NMES also evoked greater torque than at very short muscle lengths (p=0.002, CI 95%: -2.47, -0.57). The shortest quadriceps muscle length generated the highest perceived discomfort for a given current amplitude. The amount of fatigue was greater when muscle length allowed greater torque generation in the pre-fatigue condition. Strength gains (isometric and isokinetic) were greater for a protocol applied at the optimal muscle length than for short. The quality of evidence was very high for all the comparisons for evoked torque. Thus, new findings are not expected to alter the conclusions presented in this review. However, more studies are necessary for certainty on the other outcomes. Optimal muscle length should be considered the primary choice during NMES interventions, as it promotes higher levels of force production and facilitates the preservation/gains of muscle force and mass with reduced perceived discomfort. However, a longer than optimal muscle length may also be used due to possible muscle lengthening at high evoked tension. A thorough understanding of these physiological principles is imperative for the appropriate prescription of NMES for healthy and clinical populations.

Keywords: Electrophysical agents; Dynamometry; Fatigue; Sensory discomfort; Physical Rehabilitation

**Introduction**

Neuromuscular electrical stimulation (NMES) can improve neuromuscular function in different populations (1-3). NMES-related outcomes may be influenced by several parameters, such as device-derived parameters (e.g., current type, current amplitude, stimulation frequency, pulse duration) (3-8), device-human interface (e.g., electrode type, size, and configuration; nerve or muscle stimulation), and human-derived parameters (e.g., type of contraction, target muscle, muscle-tendon unit length, health status) (9-15). These factors should interact to produce greater evoked contractions with minor discomfort to obtain maximum NMES-efficiency. Accordingly, muscle length is a specially recognized modulator of the force generation capacity of muscle fibers and the stimulus for strengthening and hypertrophy when it comes to voluntary exercise (16-19). As such, the optimal muscle length (or optimal, or preferable) for NMES, would be at the plateau region of the force-length relationship, by manipulating joint angle (20-22). However, to date, there is no summary of scientific data on how NMES effects can be optimized by the “best joint angle”, which may limit the benefits of NMES-based programs and novel approaches to mitigate perceived discomfort and fatigue.

The muscle force-length relationship of single muscle fibers *in vitro* is classically known to display an ascendant and a descending limb, with a plateau in between, where the maximum tension is achieved (20), and has been further supported by recent research (23). Moreover, *in vivo,* experiments must account for the moment arm for each muscle because the joint angle-torque relationship becomes the practical translation of the force-length relationship for assessing human performance (24). The early study of Marsh et al. (25) brings relevant data showing the different shapes of the torque-angle relationship of the dorsiflexors according to the type of activation (MVC or stimulated at different frequencies). However, while the dorsiflexors show progressively greater evoked torque as the ankle angle is more towards plantarflexion (25), for the quadriceps muscle, the torque is greater closer to 60º of knee flexion considering a range from 30º to 90º (14). These divergent findings can likely be attributed to a multitude of factors, including differences in moment arms, the overall joint range of motion, muscle architecture, and the characteristics of the in-series elastic tendinous tissues (9, 13, 21).

When applying NMES, it is essential not only to consider torque generation but also to minimize perceived discomfort. A narrative review (1) informed that a shortened position (e.g., extended knee for knee extensor stimulation) could lead to painful cramp-like contractions. Therefore, during the application of NMES, in addition to biomechanical considerations, alterations in muscle length may contribute to varying levels of perceived discomfort. Conversely, it is recommended that NMES training at the 'ideal' muscle length (i.e., at the joint angle that allows the greatest evoked torque generation) and subsequently progress towards a longer muscle length. Despite the absence of evidence indicating that this approach diminishes discomfort during NMES sessions, it may serve to mitigate the heightened risk of early muscle damage associated with longer muscle lengths (6).

It has been claimed that NMES at a shortened position (full knee extension) for strengthening the quadriceps promotes greater joint soft tissue protection after anterior cruciate ligament repair (26). However, according to the authors, the strength gains observed were lower than expected, considering previous studies that used a knee flexion angle of 60º (26, 27). These results corroborate those of a previous study (28) that reported a greater strengthening effect for an elongated position (65º of knee flexion) compared to a shortened position (15º of knee flexion) (24). A previous study found that NMES-induced quadriceps fatigue depends on muscle length changes due to hip and knee joint angles (12). Fatigability is greater at a knee flexion of 60° compared to 20°. In the case of a 60° knee flexion, a supine position leads to earlier fatigue, while with a 20° knee flexion, a supine position delays fatigue compared to a seated position (9). While abundant data exists on the influence of muscle length on NMES-evoked force, there has not been a systematic compilation in a review to identify the optimal muscle length for enhancing muscle adaptations and reducing NMES-induced contraction fatigue and perceived discomfort during NMES rehabilitation and training programs.

This review, therefore, was developed to summarize the research comparing different muscle length settings during NMES following the Cochrane collaboration recommendations (29) to assess the effects of these interventions on outcomes important for NMES-based programs. Specifically, we compared the effects of muscle length on NMES-evoked isometric torque, contraction fatigability, discomfort, and strength training adaptations in healthy and clinical adult populations. We hypothesized that the optimal muscle length, according to the preferable joint angle for maximum force development, would induce greater fatigue, mainly due to the greater absolute muscle force (and torque) at the fresh (pre-fatigue) state. We also hypothesized that the discomfort would be lower at the optimal muscle length during NMES contractions. Finally, we hypothesized that strength gains would be more pronounced at the optimal muscle length, while greater hypertrophy could potentially be attained through more elongated positions in comparison to the optimal muscle length.

**Material and Methods**

This systematic review followed the recommendations proposed by the Cochrane Collaboration and the Preferred Reporting Items for Systematic Reviews and Meta-Analyses Statement (PRISMA) (29, 30), and was registered at PROSPERO (CRD42022332965). The Supplemental Digital Content 1 provides a PRISMA checklist.

*Search strategy*

We searched seven electronic databases: PUBMED, Web Of Science, EMBASE, PEDro, BIREME, SCIELO, and Cochrane, from June 2022 to October 2023. The search strategy was established following the PICO strategy (Population, Intervention, Control, Outcome) for any adult population (≥ 18 years old, healthy or clinical) submitted to electrical stimulation applied at different muscle lengths (according to joint angle) and that evaluated its effects on maximum evoked torque, contraction fatigability, perceived discomfort, or strength training adaptations.

We used the following descriptors in our search strategy, without restrictions on language or date of publication: “healthy”, “adults”, “participants”, “volunteers”. “muscle weakness”, “muscle atrophy”, “cachexia”, “elderly”, “muscle diseases”, “muscle paralysis”, “Parkinson”, “neuromuscular disease”, “stroke”, “multiple sclerosis”, “anterior cruciate ligament reconstruction”, “chronic obstructive pulmonary disease”, “lung disease”, “cardiac disease”, “obese”, “vascular disease”, “diabetes”, “orthopedic patients”, “nephrology patients”, “electric stimulation”, “functional electrical stimulation”, “neuromuscular electrical stimulation”, “muscle length”, “joint angle”, “joint position”, “torque”, “force”, “fatigue”, “discomfort”, “pain”. The searches were adapted for each database to identify all relevant articles. Additional articles were screened on the reference list of included studies. The search strategy for each database is described in Supplemental Digital Content 2. The grey literature was not searched due to the lack of a peer review or rigorous independent scientific review, which can produce bias and confounding factors that are not adequately addressed, while a lack of scientific rigor can significantly reduce the validity of the results.

The inclusion criteria were: i) cross-sectional, repeated measures, or randomized controlled trials; ii) electrically induced muscle contraction as an intervention to generate maximum torque, contraction fatigability, perceived discomfort, or strength training adaptations; iii) comparison of different muscle lengths (i.e., joint angles); iv) reported results that could be converted to a standardized effect (31); v) healthy or clinical adult populations (≥ 18 years old). Studies were excluded if they investigated the vocalization, respiratory, or pelvic floor muscles.

*Data extraction*

Two researchers (J.G.T.C and V.H.R) independently evaluated the titles of all articles found by the search strategy. If the title was clearly on a topic not relevant for the present review, it was excluded. In the case of uncertainty, it was selected for abstract reading, along with those that could possibly be included. The same procedure was performed during abstract reading, i.e., studies whose abstracts did not provide sufficient information regarding the inclusion and exclusion criteria were selected for full-text evaluation. Disagreements among reviewers were resolved by consensus, and if conflict persisted, a third reviewer (J.L.Q.D.) was consulted. The data extraction was performed independently by the same two reviewers via a standardized form to gather information on participants (healthy status, age, anthropometrics), interventions (joint angle comparisons, electrode characteristics and placement, electrical current parameters), and outcomes were collected. No automation tool was used.

For meta-analyses, when multiple data were available for maximum evoked torque measurements, we always chose the data expected to produce greater torque (e.g., doublet instead of twitch-evoked torque, or 50 Hz instead of 20 Hz of stimulation frequency), and we always chose data not explicitly affected by previous stimulus (e.g., non-potentiated instead of potentiated). For fatigue, we were interested in the evoked torque decline during a fatiguing protocol, as well as the evoked and or voluntary torque obtained before and after the fatiguing protocol. When the data where not available in tables or in the results section, we extracted from the graphs using the ImageJ software (v. 1.46; National Institutes of Health, Bethesda, Maryland, USA).

*Risk of Bias Assessment*

The risk of bias was assessed using the Revised Cochrane risk-of-bias tool for cross-over trials (RoB-CO) and the Physiotherapy Evidence Database (PEDro) scale. The RoB-CO assesses six domains: Bias arising from the randomization process; Bias arising from period and carryover effects; Bias due to deviations from intended intervention; Bias due to missing outcome data; Bias in measurement of the outcome; and Bias in selection of the reported result. The PEDro scale has 11 items that involve: 1) eligibility criteria (not used to calculate score); 2) random allocation; 3) concealed allocation; 4) baseline comparability; 5) blinded subjects; 6) blinded therapists; 7) blinded assessors; 8) adequate follow-up; 9) intention-to-treat analysis; 10) between-group statistical comparisons; 11) point estimate and variability. Each item was marked as "yes (1/0)" or "no (0/0)" and provided a 0 to 10 scale.

### Quality of Evidence

The Grading of Recommendations, Assessment, Development, and Evaluation (GRADE) was used to assess the overall quality of evidence. GRADE has 5 domains: Study design and risk of bias; Inconsistency; Indirectness; Imprecision, and Other factors (e.g., reporting bias, publication bias). Regarding classification, high quality of evidence means consistent results in at least 75% of the clinical trials of good methodological quality, presenting consistent, direct, and precise data with no suspicious or known publication bias, and further research is unlikely to alter the estimate or the confidence in the results. Moderate quality of evidence means that at least one domain is not met, and new research is likely to significantly impact the confidence in the effect estimate. Low-quality evidence means that 2 of the domains are not met, and further research is expected to significantly impact the confidence in the effect estimate and is likely to alter the estimate. Very low-quality evidence means that 3 domains are not met, and the results will be highly uncertain (32).

*Data Analysis*

Meta-analyses were performed using the Software Review Manager 5.4.1 (The Cochrane Collaboration) if the available data were sufficient and at least two studies could be fairly compared, i.e., by matching main characteristics: population, muscle involved, and muscle length. Considering these basic rules, there was compatibility for meta-analyses only for the evoked torque, but not for perceived discomfort, fatigue, and strength training adaptations. The continuous values (mean and standard deviation) of maximum evoked torque, and the number of participants for each group comparison were extracted to estimate the standardized mean difference (SMD) of the intervention and its 95% confidence interval (CI) using a random-effects model with inverse variance as a statistical method. The test for overall effect (Z-test) provided the p-value. The level of significance was set at p < 0.05.

Reference to joint angle may change; for example, the full knee extension is more commonly cited as 0º, although it may also be considered 180º. Therefore, we have defined full extension as 0° for the knee and hip joints. When discussing other joints, we provide the reference angle as needed. To enhance study compatibility for meta-analysis, we categorized the quadriceps muscle length (with a fixed hip angle set between 70° and 90°) based on the knee angle as follows: very short (0° - 30°), short (31° - 54°), optimal (55° - 65°), long (66° - 90°), and very long (> 90°). Most studies use round numbers to specify knee joint angles, although some studies involving the ankle and elbow joints have employed specific angles (e.g., 16°, 48°, 104°).

A gold standard standard for classifying quadriceps muscle length is currently lacking. However, in most studies, a range between 55° to 65°, with 60° being the common midpoint, is often considered as the optimal or intermediate length (9, 33, 34). The terms 'short' and 'long' are typically defined within the context of specific joint angles being compared. For instance, Skurvydas et al. (35) defined 'short' as a knee flexion angle of 50° and 'long' as 100°, while Place et al. (36) used 'short' for 35° and 'long' for 75° of knee flexion. In contrast, Rassier (33) described 'optimal' as 60°, 'shorter than optimal' as 30°, and 'longer than optimal' as 90°. To perform a meta-analysis, we ensured that the stimulated muscles, participant groups (comprising healthy and young individuals), and the methodology for torque generation (including nerve or motor point stimulation) were consistent across the studies.

**Results**

***Search findings***

Our search strategy retrieved 1185 records. After removing 277 duplicates and 82 clinical trial registries, we screened the titles of 826 studies. Titles that were not eligible were excluded (n = 761). Thereafter, 63 studies were screened through abstract, with 36 studies remaining for full-text screening. After full-text reading, six studies were excluded (one standardized the same percentage of MVC during evoked torque (not allowing any comparison of maximum evoked torque), one did not assess isometric torque, one did not specify joint angles, one did not show any result that could allow joint angle/muscle length comparison, one showed the same variable of our interest with the same population of another study from the same authors, and one was in Japanese language. Thus, 30 studies were selected and we also included six additional studies. One study was found on a reference list (33), two were found by manual search on Google (34, 35), two were published by some authors of this systematic review (12, 36), and another was provided by an author upon request (37). Consequently, a total of 36 studies were in this review. The PRISMA flowchart of study identification and selection is presented in Fig 1.

Figure 1. Flowchart for identifying and selecting articles for final inclusion (based on the Prisma flowchart template).

***Quality of evidence***

The results of the RoB-CO assessment can be found in Supplemental Digital Content 3. In brief, none of the studies exhibited bias resulting from deviations in the intended intervention, missing outcome data, outcome measurement issues, or selective result reporting. While one study drew attention to potential carryover effects, three studies did not provide clear reporting on their randomization process. Moreover, ten studies raised concerns due to high risk or potential issues related to carryover effects. The GRADE assessments are provided along with the description of the meta-analyses results.

The results of the PEDro scale are shown in Table 1. Three studies scored ‘7’, 22 studies scored ‘6’, eight studies scored ‘5’, and 3 studies scored ‘4’.

| **Table 1.** Methodological quality of included articles (PEDro scale). | | | | | | | | | | | |
| --- | --- | --- | --- | --- | --- | --- | --- | --- | --- | --- | --- |
| **Author (Year)** | **Random allocation** | **Concealed allocation** | **Similar groups baseline** | **Subject blinding** | **Therapist blinding** | **Assessor blinding** | **Adequate follow-up** | **Intention-to-treat analysis** | **Between-group comparisons** | **Point estimate & variability** | **Total** |
| **Cavalcante et al., 2022** | Y | Y | Y | Y | N | N | Y | N | Y | Y | 7 |
| **Cavalcante et al., 2021a** | Y | Y | Y | Y | N | N | Y | N | Y | Y | 7 |
| **Harnie et al., 2020** | Y | Y | Y | N | N | N | Y | N | Y | Y | 6 |
| **Hali *et al*., 2020** | Y | Y | Y | N | N | N | N | N | Y | Y | 5 |
| **Fouré et al., 2019** | Y | Y | Y | N | N | N | N | N | Y | Y | 5 |
| **Debenham & Power, 2019** | N | N | Y | N | N | N | Y | N | Y | Y | 4 |
| **Scott et al., 2019** | Y | Y | Y | Y | N | N | Y | N | Y | Y | 7 |
| **Gavin et al., 2018** | Y | Y | Y | N | N | N | Y | N | Y | Y | 6 |
| **Merlet et al., 2018** | Y | Y | Y | N | N | N | Y | N | Y | Y | 6 |
| **Yanase et al., 2017** | Y | Y | Y | N | N | N | Y | N | Y | Y | 6 |
| **Visscher et al., 2017** | Y | Y | Y | N | N | N | Y | N | Y | Y | 6 |
| **Bampouras et al., 2017** | Y | Y | Y | N | N | N | N | N | Y | Y | 5 |
| **Ando et al., 2016** | Y | Y | Y | N | N | N | Y | N | Y | Y | 6 |
| **Bremner et al., 2015a** | Y | Y | Y | N | N | N | Y | N | Y | Y | 6 |
| **Bremner et al., 2015b** | Y | Y | Y | N | N | N | Y | N | Y | Y | 6 |
| **Frigon et al., 2011** | Y | Y | Y | N | N | N | Y | N | Y | Y | 6 |
| **Skurvydas et al., 2010** | Y | Y | Y | N | N | N | N | N | Y | Y | 5 |
| **Marion et al., 2009** | Y | Y | Y | N | N | N | Y | N | Y | Y | 6 |
| **Ruiter et al., 2008** | Y | Y | Y | N | N | N | Y | N | Y | Y | 6 |
| **Kooistra et al., 2007** | Y | Y | Y | N | N | N | Y | N | Y | Y | 6 |
| **Lee et al., 2007** | Y | Y | Y | N | N | N | Y | N | Y | Y | 6 |
| **Guerrits et al., 2005** | Y | Y | Y | N | N | N | Y | N | Y | Y | 6 |
| **Miyamoto & Oda, 2005** | Y | Y | Y | N | N | N | N | N | Y | Y | 5 |
| **Kooistra et al., 2005** | Y | Y | Y | N | N | N | N | N | Y | Y | 5 |
| **Place et al., 2004** | Y | Y | Y | N | N | N | Y | N | Y | Y | 6 |
| **Ruiter et al., 2004** | Y | Y | Y | N | N | N | Y | N | Y | Y | 6 |
| **Babault et al., 2003** | Y | Y | Y | N | N | N | Y | N | Y | Y | 6 |
| **Maffiuletti et al., 2003** | Y | Y | Y | N | N | N | Y | N | Y | Y | 6 |
| **Hansen et al., 2003** | Y | Y | Y | N | N | N | N | N | Y | Y | 5 |
| **Mela et al., 2001** | Y | Y | Y | N | N | N | N | N | Y | Y | 5 |
| **Rassier, 2000** | Y | Y | Y | N | N | N | Y | N | Y | Y | 6 |
| **Sacco et al., 1994** | N | N | Y | N | N | N | Y | N | Y | Y | 4 |
| **McNeal & Bake, 1988** | N | N | Y | N | N | N | Y | N | Y | Y | 4 |
| **Fitch & McComas, 1985** | Y | Y | Y | N | N | N | Y | N | Y | Y | 6 |
| **Fahey et al., 1984** | Y | Y | Y | N | N | N | Y | N | Y | Y | 6 |
| **Marsh et al., 1981** | Y | Y | Y | N | N | N | Y | N | Y | Y | 6 |

***Included studies main characteristics***

Table 2 outlines the key characteristics of the studies included in our analysis. These studies were published between 1981 and 2022, written in English, and were categorized as employing a repeated measure design (crossover), randomized clinical trial, or self-control (where opposite limbs were compared) approach. A total of 448 healthy young participants, ranging in age from 19 to 40 years, were enrolled across these studies. Their contributions were aimed at assessing various aspects, including maximum evoked torque (n = 268), contraction fatigability (n = 87), discomfort (n = 82), and muscle strengthening (n = 22). Moreover, six patients with spinal cord injuries were included and paired with an able-bodied sample (14), and one study dealt only with a healthy old sample of 15 participants (38). 23 studies reported a measure of maximal electrically induced torque (peak twitch, supramaximal triplet stimulation, tetanic torque, or evoked torque), eight studies applied an NMES-fatiguing protocol, five studies reported the perceived discomfort, one study assessed strength gains (28), and two studies assessed other strength outcomes (without reporting only torque): the rate of torque development (39) and the extra forces with preceding activity (40).

| **Table 1** - Characteristics of the included studies. | | | | | | |
| --- | --- | --- | --- | --- | --- | --- |
| **Authors** | **Sample Size/ % male** | **Sample Characteristics** | **Electrode number (size), and type** | **Electrodes Position** | **Joint angles** | **Outcomes** |
| **Cavalcante et al., 2022** | 20/100% | Healthy; age: 24±4.6 y; weight: 77±9.3 kg; height: 177.6±6.3 cm | 4 (5 x 5 cm) self-adhesive | Right quadriceps muscle bellies | Hip: 0º or 85º  Knee: 20º or 60º | Fatigue |
| **Cavalcante et al., 2021** | 20/100% | Healthy; age: 24±4.6 y; weight: 77±9.3 kg; height: 177.6±6.3 cm | 4 (5 x 5 cm) self-adhesive | Right quadriceps muscle bellies | Hip: 0º or 85º  Knee: 20º or 60º | Evoked torque  Discomfort |
| **Harnie et al., 2020** | 32/72% | Healthy; age: 22.1±3.2 y; weight: 67.5±10.3 kg; height: 174.7±8.2 cm | cathode (0.5 cm diameter)  1 (5 x 9 cm) | Right leg; femoral triangle, 3–5 cm  below the inguinal ligament and gluteal fold | Hip:  Knee: 90º or 30º | Peak twitch torque |
| **Hali *et al*., 2020** | 10/100% | Healthy; age: 24±3 y; weight: 81±7 kg; height: 181±5 cm | Not informed | Non-dominant leg (left), tibial nerve | Hip: 90º  Knee: 90º  Ankle: 20º DF or 20º PF | Peak twitch torque |
| **Fouré et al., 2019** | 10/60% | Healthy; age: 27±4 y; weight: 63.5±9.2 kg; height: 173±10 cm | 1 (5 x 10)  2 (5 x 5) | Proximal thigh: ~5cm below inguinal ligament) VL and VM muscle bellies | Hip: ~90º  Knee: 50º or 100º | Fatigue |
| **Debenham & Power, 2019** | 8/100% | Healthy; 24 ± 3 y; weight: 72±11kg; height: 177±9 cm | Anode: ECG electrode  Cathode: (6–8x8–10 cm) made aluminum electrode pad wrapped in a damp paper towel covered in conductive gel | Anode: inguinal triangle  Cathode: inferior gluteal fold. | Hip: Not informed  Knee: 35º and 100º | Rate of Torque Development |
| **Scott et al., 2019** | 18/50% | Healthy; age: 24.7±5.9 y; weight: 78.5±13.2 kg; height: 173.3±11 cm | 2 (7.62 x 12.7 cm) | Right proximal and distal quadriceps muscles | Hip: Seated  Knee: 30, 60 or 90 | Evoked torque |
| **Gavin et al., 2018** | 15/46.7% | Healthy old; 66 ± 8 y; weight: 73.0 ± 14.1; height: 168.3 ± 8.2 cm | Single device | Common peroneal nerve (muscles: peroneus longus and tibialis anterior) | Hip: ~90º Knee: 0º, 45º, 90º | Discomfort |
| **Merlet et al., 2018** | 12/58% | Healthy; age: 22.5±1.2 y; weight: 63.5±9.2 kg; height: 172.5±9.7 cm | 1 (1.0 cm diameter)  1 (5 x 10 cm) | Right posterior tibial nerve | Hip: 70º  Knee: 0º, 30º, 90º  ankle: 90º | Peak twitch torque |
| **Yanase et al., 2017** | 40/100% | Healthy; age 24.4 ± 3.6 y; weight: 67.3 ± 7.6; height: 172.1 ± 5.6 cm | Bipolar electrodes, 5 x 5 cm | Right or left shoulder; one electrode over the motor point of the infraspinatus, other over the muscle belly. | maximal internal rotation (IR; 82.5° ± 9.6°); neutral rotation (NEUT); max external rotation (ER; 86.1° ± 13.5°), | Muscle swelling and soreness |
| **Visscher et al., 2017** | 16/100% | Healthy; age: 26±4 y; weight: 78±6 kg; height: 182±5 cm | 1 (5 cm diameter)  1 (5x10 cm) | Femoral nerve trunk (individual muscle bellies stimulation omitted) | Hip: 90º  Knee: 30º, 65º, 90º | Peak twitch torque |
| **Bampouras et al., 2017** | 9/100% | Healthy; age: 30.2±7.7 y; weight: 81.7±11.2 kg; height: 178±0.09 cm | 2 (7 x 12.5 cm) | Proximal and distal regions of the quadriceps muscle group  with the cathode being the proximal electrode. | Hip: 90º or 160º  Knee: 90º | Tetanic torque |
| **Ando et al., 2018** | 8/100% | Healthy; age: 24±2 y; weight: 63.5±9.2 kg; height: 172.5±9.7 cm | one cathode (2.0 x 3.5 cm) and two anodes (7 x 10 cm) | Femoral nerve | - Hips: 70° of flexion (0º is anatomical position)  - Knee: 60° (extended) and 110° (flexed) | Fatigue |
| **Bremner et al., 2015a** | 16/0% | Healthy; age: 21.5±2.4 y; weight: 67.7±7.7 kg; height: 162.4±5.3 cm | 4 (5x9 cm) | Right leg; electrodes were placed over the femoral  nerve and the motor points of each of the superficial  quadriceps muscles | Hip: 85º  Knee: 15º or 60º | Normalized peak torque |
| **Bremner et al., 2015b** | 20/50% | Healthy; age: 21.3 ± 2.1 y; weight: 75.6 ± 15.3; height: 167.4 ± 8.5 cm | Four 5 × 9 cm | Over the proximal and distal VL, proximal RF, and distal VM motor points. | Hip: 85º Knee: 15º or 60º | Discomfort |
| **Frigon et al., 2011** | 14/64.28% | Healthy; no other data available | TA: 5 cm, rounded;  GM: 7x13 cm | Proximal and distal TA; and proximal and distal GM | Hip: 90º Knee: 170 - 180º (full extension) Ankle: | Extra forces |
| **Skurvydas et al., 2010** | 11/100% | Healthy; 24.8 ± 3.7 y; weight: 78.2 ± 4.7kg; height: 179.9 ± 3.6 cm | two carbonized rubber electrodes covered gel: 6 x 11 cm (proximal) and 6 x 20 cm (distal) | Right quadriceps; transversely across the width of the proximal  and the distal  portion of the quadriceps. | Hip: seated Knee: not informed Ankle: 9º dorsiflexion, 16º plantar flexion, and 44º plantar flexion | Evoked torque |
| **Marion et al., 2009** | 8/70% | Healthy; 28.2±3.63 y | Two 7.5 x 12.5 | Right quadriceps muscle | Hips: 15º  Knee: 20º, 40º, 65º, and 90º | Fatigue |
| **Ruiter et al., 2008** | 10/100% | Healthy; age: 26.0±7.1 y; weight: 73.0±5.2 kg; height: 181.9±8.3 | Cathode: 5x5 cm Anode: 13x8 cm | Cathode: femoral nerve Anode: gluteal fold | Hip: 70º  Knee: 30º, 60º, 90º | Triplet torque |
| **Kooistra et al., 2007** | 7/100% | Healthy; age: 23-32 y; weight: 69-83 kg; height: 173-193 | Cathode: 5x5 cm Anode: 13x8 cm | Cathode: femoral nerve Anode: gluteal fold | Hip: 70º  Knee: 30º, 60º, 90º | Triplet torque |
| **Lee et al., 2007** | 5/60% | Healthy; age: 22.5±1.2 y; weight: 70.2±12 kg; height: 172.9±7.3 | - | Right quadriceps muscle | - Hips:75° of flexion  - Knee: 15º and 90º | Fatigue |
| **Guerrits et al., 2005** | 8/- | Healthy; age: 37±4 y; weight: 78±3 kg; height: 183±2 | 1 (5 x 5 cm)  1 (8 x 13 cm) | Femoral nerve (cathode) and medial part of the quadriceps muscle (anode) | Hip: 70º  Knee: 30 to 90 (10º step) | Triplet torque |
| **Miyamoto & Oda, 2005** | 9/100% | Healthy; 24.5±1.1 y; weight: 68.1±2.6 kg; height: 171.3±2.8 cm | Carbon-impregnated rubber electrodes (3.5·2 cm) with conductive gel and secured to the right upper arm with adhesive tape | One electrode was placed on the motor point of the biceps brachii, and the other was placed on the distal portion of the bíceps brachii. | Elbow: 75, 90, 105, 120, 135, and 150 (full extension = 180). | 30 Hz Tetanic torque;  Peak Twitch torque |
| **Kooistra et al., 2005** | 8/100% | Healthy; 25.0 ± 3.7 | Cathode: 5 x 5 Anode: 13 x 8 | Cathode: femoral nerve Anode: gluteal fold | Hip: 70º Knee: 30º, 60º, and 90º | Triplet torque |
| **Place et al., 2004** | 11/100% | Healthy; 24±4 y; weight: 67±7 kg; height: 177±6 cm | Cathode: monopolar ball electrode 0.5 cm diameter  Anode: 10 x 5 | Cathode: femoral nerve Anode: gluteal fold | Hip: 90º  Knee: 35º, 75º | Peak twitch torque |
| **Ruiter et al., 2004** | 7/100% | Healthy; 19-40 y; weight: 79-85 kg; height: 172-194 cm | Cathode: 5 x 5 Anode: 13 x 8 (?) | Cathode: femoral nerve Anode: gluteal fold | Hip: 70º (?)  Knee: 30º, 60º, 90º | Octet |
| **Babault et al., 2003** | 9/100% | Healthy; age: 20.6±1.6 y; weight: 73.8±6.3 kg; height: 177.3±5.7 | Cathode: ball probe (100 mm diameter) Anode: 10 x 5 cm | Cathode: femoral nerve Anode: between the greater trochanter and the inferior iliac crest. | Hip: 90º  Knee: 35º, 55º, 75º | Peak twitch torque |
| **Maffiuletti et al., 2003** | 11/100% | Healthy; age: 26.4±5.4 y; weight: 71.4±8.8 kg; height: 177.5±6.8 | 1 (0.5 cm diameter)  1 (5 x 10 cm) | Right leg; femoral triangle, 3–5 cm  below the inguinal ligament and gluteal fold | Hip: 0º or 90º  Knee: 90º | Peak twitch torque |
| **Hansen et al., 2003** | 13/ 53.8% | Healthy; 26.1±3.2 y; weight: 68.57±0.6 kg; height: 173.9±8.7 cm | two electrodes; size not mentioned | Biceps braquialis motor point | Shoulder: not informed  Elbow: 48 , 62 , 76 ,  90 , 104 , 118 , 132 , 146 , and 160 | Doublet twitch torque |
| **Mela et al., 2001** | 6/66.6% | Healthy; age: 30.3±6.8 y; | cathode: circular (1.5-cm radius); indifferent: 5x9 cm | Cachode on deep peroneal nerve; indifferent electrode on distal bony part of the shank | Hip: seated Knee: not informed Ankle: 9º dorsiflexion, 16º plantar flexion, and 44º plantar flexion | Peak twitch torque |
| **Rassier, 2000** | 10/- | Healthy; - | 2 (13 x 12 cm) | Right quadriceps muscle (proximally and distally) | "Hip: seated  Knee: 30º, 60º, 90º" | Twitch torque  Fatigue |
| **Sacco et al., 1994** | 14/- | Healthy; age: 34 (25 – 59 y). | Two 4-cm-diam | TIbialis anterior | Knee: Not informed  Ankle: 100º (optimum) and 80º (short) | Fatigue |
| **McNeal & Bake, 1988** | 10/50% | Healthy; age: 23-33 y. | 1 (4 x 5 cm)  1 (4 x 9 cm) | Left quadriceps muscle bellies | Hip: 60  Knee: 15º, 45º, 75º | Evoked torque |
| **Fitch & McComas, 1985** | 10/- | Healthy; 31.1 ± 8.5 | rectangular pieces of aluminum foil (2 x 4 cm); | Cathode over peroneal nerve; anode over the anterior-superior aspect of tibialis anterior | 15º and 25º of dorsiflexion | Fatigue |
| **Fahey et al., 1984** | 55/50.90% | Healthy; - | Two 5.1 x 10.2 | Quadriceps muscle bellies | Hip: ? Knee: 0º or 65º | Strengthening for isometric and isokinetic torque |
| **Marsh et al., 1981** | 5/100% | Healthy; 19- 37 y | Cathode: oval lead plate 5 x 2, covered by saline-soaked cloth);  anode: cloth pad soaked in saline and mounted on a steel plate (8 x 8) | Cathode: upper part of TA; Anode: the lower third of TA. | 11 angles (from 30º PF to 20º DF), in steps of 5º | Tetanic stimulation at 40 Hz |

DF: dorsiflexion; PF: plantar flexion; (?) presumed considering other articles by the authors; *

***Physical parameters of the electrical stimulation protocols***

All parameters used in the interventions are presented in Table 3. The current type and waveform were usually not fully described, and only two studies reported both parameters. Pulse duration ranged between 50 and 1000 μs, but 2 studies did not report pulse duration. When a tetanic contraction was applied in evaluation and/or intervention, studies (n = 4) used stimulation ranging from 75 Hz to 300 Hz, with a pulse duration ranging from 200 μs to 600 μs. Notably, one study deviated from the use of pulsed current and opted for the utilization of the Russian current.

Stimulated muscles and joint angles are presented in Table 2. The quadriceps femoris muscle was the more commonly studied muscle, followed by the ankle plantar flexors and dorsiflexors, and the elbow flexors. Only one study investigated an external rotator (infraspinatus) of the shoulder.

| **Table 2** - NMES Parameters used in the included studies. | | | | | | | | |
| --- | --- | --- | --- | --- | --- | --- | --- | --- |
| **Author** | **Current Type** | **Waveform** | **Pulse Frequency (Hz)** | **Phase Duration (μs)** | **Time ON/OFF (sec) or Duty Cycle** | **Additional parameters** | **Fatigue protocol duration** | **Intensity** |
| **Cavalcante et al., 2022** | Bipolar | - | 100 | 500 | 10 s / 120 s | - | Does not apply | MEIC (EVA 8/10) |
| **Cavalcante et al., 2021** | Bipolar | - | 100 | 500 | 10 s / 120 s | - | 12 contractions | MEIC (EVA 8/10) |
| **Harnie et al., 2020** | Constant | Rectangular | Single pulse | 1000 | Does not apply | 400 V | Does not apply | 120% supramaximal (84.4 ± 15.7 mA) |
| **Hali et al., 2020** | - | Square | Single pulse | 200 | Does not apply | 400 V | Does not apply | 20% supramaximal stimulation |
| **Fouré et al., 2020** | Biphasic symmetric | Rectangular | 100 | 400 | 5 s / 35 s | - | 40 contractions | MEIC |
| **Debenham & Power, 2019** | - | Square | 300 Hz (Octect) | Single pulse: 1000; octet: 200 | - | 400 V | Does not apply | single pulse: 15% above plateau  octet: plateau (maximum torque) |
| **Scott et al., 2019** | - | Square | 75 | 600 | 4 s / 60 s | - | Does not apply | MEIC (EVA 7/10) |
| **Gavin et al., 2018** | - | - | Single pulse | 400* | - | - | Does not apply | 27 mA |
| **Merlet et al., 2018** | - | Rectangular | Single pulse | 1000 | - | 400 V | Does not apply | 120% twitch force |
| **Yanase et al., 2017** | - | - | 20 | 250 | 5 s / 2 s | - | 20 min (fatigue not assessed) | Maximum tolerated |
| **Visscher et al., 2017** | - | Rectangular | Single pulse | 1000 | - | - | Does not apply | 100 mA |
| **Bampouras et al., 2017** | Bipolar | - | 100 | 200 | 1 s/- | 10 ms IPI | Does not apply | Supramaximal (512 ± 124.6 mA) |
| **Ando et al., 2018** | Constant | - | 20 | 200 | 70 s / 0 s | 400 V | 1 contraction | 120% twitch force (36 – 134 mA) |
| **Bremner et al., 2015a** | Russian |  | Carrier frequency:2500 | Not informed | / 120 s | Burst frequency: 50 bursts/s | Does not apply | Not clear |
| **Bremner et al., 2015b** | Russian | - | Carrier frequency: 2500 | Not informed | 15 s (single contraction) | Burst frequency: 50 bursts/s | Does not apply | 30% - 40% MVC |
| **Frigon et al., 2011** | - | - | 25 and 100 | 1000 | - | - | Does not apply | 10-15% of Maximal evoked twitch at an ankle joint of 90° for plantarflexors, and at 120° for dorsiflexors |
| **Skurvydas et al., 2010** | - | Square | 100* | 500 | 1 s / 10 s | - | Does not apply | 10% supramaximal |
| **Marion et al., 2009** | Constant | - | 33 | 600 | 1 s / 1 s | - | 78 contractions | 20% MVC at 90º of knee flexion |
| **Ruiter et al., 2008** | Constant | - | 300 Hz (triplet) | 200 | Does not apply | Does not apply | Does not apply | 50 mA over maximum at 90º of knee flexion (250 a 400 mA). |
| **Kooistra et al., 2007** | - | Rectangular | 300 Hz (triplet) | 100 | Does not apply | Does not apply | Does not apply | 50 mA over maximum at each angle |
| **Lee et al., 2007** | - | - | 40 | 600 | Not clear | - | 120 contractions | 20% MVC |
| **Guerrits et al., 2005** | - | Square | 300 Hz (triplet) | 200 | / 120 s | - | Does not apply | Supramaximal |
| **Miyamoto & Oda, 2005** | - | Rectangular | 30* | 100 | - | - | Does not apply | maximal twitch contraction |
| **Kooistra et al., 2005** | - | Rectangular | 300 (triplet) | 100 | - | - | Does not apply | 50 mA over maximum torque (procedure at 60º, but used for the other positions. i.e., same amplitude for all positions. |
| **Place et al., 2004** | Constant | - | Does not apply | 1000 | Does not apply | 400 V | Does not apply | Maximum ( 300 – 500 mA) |
| **Ruiter et al., 2004** | Constant | - | 300 Hz | 100 | Does not apply | Does not apply | Does not apply | Maximum |
| **Babault et al., 2003** | - | Square-wave | Does not apply | 1000 | Does not apply | 400 V | Does not apply | 10% supramaximal (60 – 130 mA) |
| **Maffiuletti et al., 2003** | - | Rectangular | Single pulse | - | - | 400 V | Does not apply | Supramaximal (50 – 80 mA) |
| **Hansen et al., 2003** | - | Square | - | 800 | - | 8ms inter-twitch interval | Does not apply | Doublet |
| **Mela et al., 2001** | - | Rectangular | 50* | 300 | 2 s/ not clear | - | Does not apply | beneath the subject’s pain threshold |
| **Rassier, 2000** | - | Square | 50 | 50 | 5 s / 5 s | - | 9 contractions | 50% MVC |
| **Sacco et al., 1994** | - | Rectangular | 30 | - | 15 s / 5 s | - | 6 contractions | 20% MVC |
| **McNeal & Bake, 1988** |  | - | Single pulse | - | - | - | Does not apply | 60 mA |
| **Fitch & McComas, 1985** | - | Rectangular | 20 | 50 | 90s/0s | - | Single contraction | Supramaximal stimulus intensities |
| **Fahey et al., 1984** | asymmetrical bi-phasic | Square | 50 | Not informed | 10s/35s | 25 V; Pulse time to peak stimulus: 2 s | 60 contractions (15 min); fatigue not assessed | 42.6 ± 4.4 (slowly increased to the highest level comfortably tolerated) |
| **Marsh et al., 1981** | - | Rectangular | 40* | 100 | - | - | Does not apply | 20% supramaximal |
| IPI: interpulse interval; bps: bursts per second; MEIC: maximal electrically induced contraction; VAS: Visual Analogue Scale; MVC: Maximal voluntary contraction; *maximum frequency/pulse width chosen among two or more options. | | | | | | | | |

***Evoked Torque***

Meta-analyses were only possible for this outcome. Twenty-three studies reported a measure of maximum evoked contraction through motor point stimulation (tetanic contraction or twitch) or nerve stimulation (twitch, doublet, triplet, or octet) comparing the value reached in different muscle lengths. Of these, 17 were conducted on the knee extensors, two on the ankle plantar flexors, two on the ankle dorsiflexors, and two on the biceps brachialis muscle.

A total of six meta-analyses were conducted involving data from 12 studies (Fig 2 A-F) that applied NMES to the quadriceps femoris muscles (there were no matching studies for other muscle groups). The study by Guerrits et al. (14) met the criteria for inclusion in the meta-analyses, yet only the data from their able-bodied participants were used, as their sample also encompassed individuals with spinal cord injuries. Furthermore, their triplet torque data for knee flexion angles of 40º and 50º were aggregated to represent short muscle length, while the data for angles of 70º, 80º, and 90º were combined to represent long muscle length. Notably, there was a significant effect (SMD: -1.59, 95% CI 95%: -2.03, -1.15, p < 0.001) that favored optimal muscle length over very short muscle length during muscle belly stimulation (Fig 2A). This observation was drawn from three studies characterized by high-quality evidence (9, 34, 41). However, for this stimulation type, no corresponding article was available for comparing other muscle lengths.

For the femoral nerve stimulation, all outcomes were graded as high-quality evidence. There was a significant effect of greater evoked torque favoring the optimal muscle length vs. very short muscle length (SMD: -2.37, CI 95%: -3.64, -1.10, p < 0.001, Fig 2B) (14, 42-46), vs. short muscle length (SMD: -1.05, CI 95%: -1.78, -0.32, p = 0.005, Fig 2E) (14, 36), and vs. long muscle length (SMD: 0.85, CI 95%: 0.48, 1.22, p < 0.001, Fig 2D) (14, 36, 42-46). Moreover, there was a significant effect of greater evoked torque favoring the long muscle length vs. very short muscle length (SMD: -1.52, CI 95%: -2.47, -0.57, p = 0.002, Fig 2C) (14, 42-47), and vs. short muscle length (SMD: -1.05, CI 95%: -1.78, -0.32, p = 0.005, Fig 2F) (14, 36, 48).

Figure 2. Meta-analyses of (A) very short muscle length vs. optimal muscle length for maximum evoked torque produced during quadriceps muscle motor point stimulation, (B) very short muscle length vs. optimal muscle length for maximum evoked torque produced during femoral nerve stimulation, (C) very short muscle length vs. long muscle length for maximum evoked torque produced during femoral nerve stimulation, (D) optimal muscle length vs. long muscle length for maximum evoked torque produced during femoral nerve stimulation, (E) short muscle length vs. optimal muscle length for maximum evoked torque produced during femoral nerve stimulation, and (F) short muscle length vs. long muscle length for maximum evoked torque produced during femoral nerve stimulation.

***NMES-induced fatigue***

Eight studies conducted an NMES-fatiguing protocol, six on the quadriceps femoris muscle (one of them through femoral nerve stimulation), and two on the tibialis anterior muscle. More flexed knee joint angles were more fatiguing for the quadriceps: 20º vs. 60º (12), 50º vs. 100º (49), 60º vs. 110º (50), and 15º vs. 90º (51). However, in cases where the comparison involved more than two joint angles, the initial capacity for torque development played a significant role in influencing the results regarding fatigability. In the investigation by Marion et al. (52), fatigue was notably pronounced at the joint angle that exhibited the highest pre-fatigue force, while conversely, the least fatigue was observed at the joint angle characterized by a lower pre-fatigue force. In this instance, 65º of knee flexion demonstrated a greater pre-fatigue force compared to angles of 20º and 90º. The study conducted by Rassier (42), exhibited contradictory outcomes and reporting. They indicated a more substantial relative decline in torque at the most elongated position (90º, compared to 60º and 30º of knee flexion), while the textual content consistently referred to greater fatigue occurring at the most shortened muscle length. Regarding the tibialis anterior, findings suggested that NMES-induced fatigue was more prominent at the optimal muscle length in contrast to the shortened position (33, 53).

***Perceived discomfort***

Six studies reported the perceived discomfort through a numeric scale. These studies used the Visual Analogue Scale (VAS), while one study also used the Verbal Rating Scale (VRS) (38). Three studies used a VAS fixed value to achieve the maximum evoked contraction during quadriceps NMES: 7 out of 10 (41) and 8 out of 10 (9, 12). One study (37) found the current amplitude required to achieve 30% to 40% of the MVC in the more extended knee angle (15º), and used the same current amplitude for the knee at 60º (which would produce a much lower percentage of the MVC for this angle) and compared the VAS. They found that, for 15º and 60º, the VAS was 32.42 mm (CI upper: 42.09; CI lower: 23.02) and 17.11 (CI upper: 27.34; CI lower: 7.09), respectively. One study, with a healthy old population (38), chose a fixed current amplitude (27 mA) for the stimulation of the common peroneal nerve to evoke isometric dorsiflexion (ankle angle not mentioned) to compare, among other variables, the perceived discomfort in three knee angles (0º, 45º, and 90º). Pulse duration was gradually increased from 50 µs to 400 µs. At the highest pulse duration, no significant difference in perceived discomfort among knee angles was observed, as assessed using both the VAS and the Verbal Rating Scale (VRS). In another study (54), a 20-minute NMES protocol was applied to the infraspinatus muscle. The participants were divided into four groups based on joint angle and type of contraction (three for isometric and one for concentric contraction), alongside a control group. For the isometric contractions, three shoulder rotation angles were employed, with participants lying prone and shoulders abducted at 90º: maximum internal rotation (82.5° ± 9.6°), neutral rotation, and maximum external rotation (86.1° ± 13.5°). The initial five minutes of muscle stimulation were utilized to progressively increase the current amplitude to the highest tolerable level for each subject (initial VAS not reported). This amplitude remained unchanged throughout the protocol. Following the protocol, no notable disparities were found among the groups (current amplitude [mA]: 83.5 ± 20.4, 85.0 ± 27.2, and 79.0 ± 20.4; VAS [mm]: 63.1 ± 19.0, 65.9 ± 18.8, and 59.8 ± 19.9 for the internal, neutral, and external rotation groups, respectively). A limitation of the study was the absence of force output measurement (54). Regrettably, these studies could not be subjected to meta-analysis due to these limitations.

***Strengthening NMES training***

Among the studies, only one (28) investigated the impacts of NMES on muscle strengthening concerning muscle length. They trained in two joint angles: full knee extension (0º) and a flexed position (65º), while participants were in a supine posture (hip angle unspecified). The outcomes revealed that NMES led to heightened isometric and isokinetic strength in both positions. Notably, it appeared that NMES exhibited greater effectiveness in enhancing isokinetic performance when the knee was flexed during treatment.

**Discussion**

This is the first systematic review to assess the effect of muscle length on maximum electrically induced torque, perceived discomfort, NMES-induced fatigue, and strength following an NMES-strengthening program. We found that, for the quadriceps femoris muscle, greater evoked torque was obtained in the optimal muscle length compared to short and long muscle lengths. Also, the long length allowed greater evoked torque than the short muscle length. According to the GRADE recommendations (32), the quality of evidence was very high for all the muscle length comparisons during evoked torque. Thus, new findings are not expected to alter the conclusions presented in this review. Other muscles, such as the ankle dorsiflexors and biceps brachialis, also exhibit an ideal (intermediate) length. However, for the ankle plantarflexors, longer lengths (either by manipulating the ankle or the knee joint angles) allow for greater torque production, which agrees with the torque-angle relationship for this muscle group that works in the ascending limb of the force-length relation (55).

When considering a given current amplitude, a shorter length of the quadriceps muscle seems to result in increased discomfort compared to the neutral position, although no definitive conclusions can be drawn regarding other muscle lengths and different muscles. NMES-induced fatigue appears to be greater when the muscle length enables greater torque generation in the pre-fatigue condition. While protocols conducted at the ideal muscle length yield greater quadriceps strength gains compared to short lengths, there is limited data available for comparing other muscle lengths and muscles. These findings suggest that prioritizing the optimal muscle length should be the initial choice for NMES interventions, while the use of long or short muscle lengths should be carefully considered and selected based on diverse clinical factors.

***Evoked Torque***

*‘Very short’ and ‘short’ vs. ‘optimal’ quadriceps femoris muscle length*

While the force-length relationship is a well-established property of muscles, this meta-analysis marks the first instance of confirming force disparities across multiple quadriceps femoris muscle lengths during electrical stimulation. Notably, an optimal muscle length surpasses very short or short muscle lengths in its capacity to generate higher evoked torque during NMES. This fundamental discovery holds significant implications for the utilization of NMES in both clinical and athletic contexts. Consequently, endeavors to employ a shortened muscle length to facilitate a particular limb position (such as a fully extended knee) may likely constrain the potential benefits of NMES (26). The selection of muscle length should be judicious, particularly in specific scenarios such as cases involving limited/painful range of motion or non-strengthening/hypertrophy objectives. For instance, in the context of voluntary isometric knee extension exercises performed at short (43.1º ± 4.6º of knee flexion) and long (86.9º ± 6.5º) muscle lengths, the latter (long) can accelerate hypertrophy, while the former (short) yields greater strength enhancement at the training angle (56).

*‘Very short’ and ‘short’ vs. ‘long’ quadriceps femoris muscle length*

The extended muscle length demonstrated a capacity for generating higher evoked torque when compared to both very short and short muscle lengths during nerve stimulation. It is noteworthy that the established force-length relationship indicates potential alignment in force levels between short and long muscle lengths along the ascending and descending limbs of the curve (20). Indeed, it is possible to obtain greater torque at 50º of knee flexion, compared to 90º during quadriceps muscle belly stimulation (57). However, it is important to consider that long muscle length tends to induce greater tensile stress (58), a factor favorable to enhanced muscle growth (56) and improved mechanical properties of tendons (59). This, in turn, lends support to the idea of favoring long muscle lengths for prolonged utilization, aligning with long-term goals

*‘Optimal’ vs. ‘long’ quadriceps femoris muscle length*

A meta-analysis was successfully conducted, focusing only on comparing optimal and long muscle lengths during nerve stimulation. The results favored the optimal muscle length, aligning with the expectations derived from the force-length relationship. Consequently, opting for an optimal muscle length is likely to expedite progress in achieving strength gains. However, it is crucial to note that the aforementioned impact of tensile stress may render long muscle lengths more advantageous for other aspects related to muscle-tendon unit properties (58). Moreover, long muscle lengths might prompt a heightened occurrence of serial sarcomerogenesis, which holds particular value in the treatment of various clinical conditions characterized by serial sarcomere loss (such as bed-rest periods, post-surgical interventions), followed by subsequent loss of functionality (56, 59, 60).

*Other muscle length comparisons for the quadriceps femoris*

Some studies could not be meta-analyzed (35, 57, 61). Vissher et al. (61) compared the evoked torque through femoral nerve stimulation among three knee angles finding a peak twitch torque at 30º of knee flexion significantly lower than at 65º and 90º. However, the authors used a current amplitude of 100 mA for all tests without mentioning if it evoked a maximal or supramaximal stimulus. Similarly, a pioneering study (35) standardized a 60 mA amplitude for three different knee angles: 15º, 45º, and 75º during quadriceps femoris muscle belly stimulation. While similar current amplitude offers the advantage of facilitating the comparison of efficiency (i.e., the ratio of torque by current amplitude) (9), it does not inform about the maximum evoked torque.

Maffiuletti et al. (62), employing femoral nerve stimulation, and Bampouras et al. (63), using muscle belly stimulation, both observed heightened quadriceps evoked torque in the supine position compared to the seated position when the knee was fixed at 90º. This suggests that the supine posture may position the biarticular rectus femoris muscle closer to its optimal length, a concept in line with the muscle's functional range (64). However, when the knee is positioned at 60º, this difference becomes less distinct and may or may not manifest (9). An underlying explanation for these divergent outcomes might be the greater passive tension experienced by the knee extensors when the knee is flexed at 90º, compared to the 60º flexion angle. The disparities could be attributed to the variance in the knee extensor passive tension under different knee angles. Future investigations hold the potential to unveil whether the manipulation of hip angles could confer benefits in the application of NMES to knee extensors.

Care should be taken concerning definitions of short, optimal, and long muscle length. For example, Fouré et al. (49) suggested that “rehabilitation training programs including electrically induced isometric contractions should be performed at short muscle length.” However, this could mislead the conclusion that short could be better than optimal in any NMES program. However, in this study, “short” was 50º and “long” was 100º of knee flexion, and 50º is closer to the angle range commonly reported as optimal (intermediate or midrange): 55° - 65º of knee flexion (9, 36, 61).

*Length comparisons for other muscle groups*

Our research retrieved studies with other muscle length comparisons that could not be meta-analyzed. Hali et al. (65) compared a shortened position (20º plantarflexed from neutral) and a lengthened position (20º dorsiflexed from neutral) for the triceps surae muscle and found greater peak twitch torque for the long muscle length (39.5 ± 12.5 vs 11.9 ± 4.8). While the study mentioned does not include a neutral position for comparison, studies focusing on MVC have shown that greater torque is generated at lengthened muscle lengths compared to neutral positions (55, 66). This indicates that the muscle-tendon unit and joint complex being examined differ from the knee extensor mechanism in terms of their response to muscle length. Similarly, the twitch and doublet (as well as the MVC) evoked torques increase for the plantarflexors when the knee is more extended, which lengthens the biarticular gastrocnemius, although the authors seem to say in their discussion the opposite of the information expressed in their results (67).

Mela et al. (68) stimulated the dorsiflexors through the stimulation of the deep peroneal nerve, while Marsh et al. (25) applied the stimulation directly over the tibialis anterior muscle belly. Specifically for Marsh et al. (25), which assessed several joint angles (from 30° of plantar flexion to 20º of dorsiflexion in steps of 5°), the greatest torque was obtained at 10º of plantar flexion. Marsh et al. (25) may have had a misconception regarding the argument that the evoked torque didn't reach MVC values due to their stimulation method not including other dorsiflexors. In the Mela et al. (68) study, nerve stimulation applied to activate all four dorsiflexors still resulted in an average evoked torque of 50% of MVC in the respective angle position. In healthy volunteers, evoked torque rarely reaches 100% of MVC (1). Both authors, despite differing stimulation methods, consistently found that torque generation was greater in the plantarflexed (lengthened) position compared to the dorsiflexed (shortened) position. This agreement underscores the influence of joint angle on torque production, irrespective of the stimulation method's specific details.

Hansen et al. (24) assessed the force-angle relationship of elbow flexors during isometric contractions at various angles. They found that MVC, double twitch, and single twitch peak torques occurred at 90º (223.6 ± 56.3 N), 104º (223.6 ± 56.3 N), and 118º (223.6 ± 56.3 N), respectively. This indicated a rightward shift in the curves with submaximal force, possibly due to increased Ca^2+^ sensitivity with muscle lengthening during submaximal contractions (69). Future studies could explore targeting the peak region of the force-angle relationship with NMES to enhance elbow flexor strength. Utilizing submaximal curves from evoked contractions for clinical interventions is plausible given that most daily activities did not require maximal effort. Investigating this approach could optimize NMES protocols for more efficient elbow flexor strength improvement. Additionally, Miyamoto and Oda (70) observed similar findings, with torque significantly higher at 120º (or 60º, considering full elbow extension as 0º) compared to more flexed angles. Future studies may also examine the impact of shoulder and forearm angles on the elbow flexors force-angle relationship during NMES.

***Contraction fatigability***

A meta-analysis for this outcome could not be conducted due to discrepant methodologies across various studies. Notably, NMES-induced fatigue appears to intensify when the muscle length enables greater torque generation in the initial (pre-fatigue) state, which may be explained by the increased metabolic demand of higher-intensity contractions, related to more actin-myosin cross-bridge formation (33). Some studies that applied voluntary contraction-fatigue protocols are in agreement with these results (71), while others did not find significant differences (72). These studies employ varying knee joint angles and fatigue-protocol methodologies, limiting generalizability. Notably, even with increased fatigue observed after NMES-fatigue protocols, optimal muscle length continues to yield greater torque compared to shorter lengths (12, 51, 52). While the expectation that shorter muscle length generates less force, leading to reduced fatigue compared to the optimal length, holds true for the quadriceps femoris, the same principle does not uniformly apply to the tibialis anterior muscle (42). Assuming that increased mechanical loading at the muscle-tendon unit potentially leads to greater and possibly faster muscle adaptation, the higher fatigue observed at optimal length serves as evidence of the increased work induced by NMES. This underscores the importance of considering optimal length over shorter muscle lengths in long-term clinical trials. Additionally, for muscles spanning multiple joints, such as the quadriceps femoris, an added stretch, like hip extension, may accelerate the onset of fatigue and intensify its effects (12).

***Perceived discomfort***

The assessment of perceived discomfort in the context of muscle length manipulation remains somewhat limited. We could not draw strong conclusions from the available studies. While it is often reported that shorter muscle lengths can induce painful cramp-like contractions, this hypothesis has not yet been subjected to rigorous testing (1). Specifically, we found a single study evidencing that for the same current amplitude, greater discomfort is reported when quadriceps NMES is applied at 15º of knee flexion compared to 60º. These results raise considerations for dynamic contractions with NMES, once care must be taken with excessive discomfort during the final range of motion. Interestingly, studies employing the VAS as a means to achieve maximum electrically induced contractions (9, 25) have consistently yielded the same percentage of the maximum voluntary contraction. However, this may come at the cost of greater current amplitude applied at longer muscle lengths compared to shorter positions (9). Thus, a comprehensive understanding of muscle length influence on perceived discomfort holds unavoidable questions such as tracking the change in the localization of motor and sensory nerve branches under the electrodes with changes in joint angle, which may require meaningful strategies to optimize the clinical outcomes (73).

Finally, Yanase et al. (54) present a limitation as it lacks a means of torque monitoring, thereby restricting the comprehensive interpretation of their discomfort data. For a comprehensive understanding of the impact of muscle length on NMES-induced perceived discomfort, the resulting torque (indicative of contraction intensity) also needs to be taken into account. Hence, future research should emphasize the development of torque control methodologies that are adaptable to diverse clinical settings and muscles, particularly in instances where dedicated equipment like a dynamometer chair is not readily available (26).

***Strengthening by NMES training***

The effects of strength training with NMES are not fully understood yet. A solitary study (28) compared the results of a six-week NMES protocol at different knee joint angles (65º and 15º). Participants were supine during the treatment protocol, but the hip angle was not mentioned. The authors found greater strength gains for the protocol at 65º of knee flexion. Thus, we partially answered our hypothesis on muscle strengthening because no study is available for longer muscle lengths and also for hypertrophy. Despite the lack of studies, a recent review (74) on the effects of NMES on the knee post-surgery rehabilitation considers the knee angle a critical parameter that may impact functional goals. Moreover, they conclude that a 60º angle or closer would be preferable if it is clinically safe.

Most NMES protocols primarily involve isometric contractions. While isometric contractions with a fully extended knee don't require specialized equipment, they may lead to reduced strengthening gains (26). For precise control of joint angle and torque, a dynamometer chair is the optimal choice. However, such resources may be limited, particularly outside clinical settings, like home care (26). In resource-constrained clinical settings, a practical approach to managing joint angles during isometric contractions is using knee support with an ankle weight. This approach, demonstrated by Toth et al. (75), effectively maintains the knee at a set angle, e.g., 40°. Another viable strategy involves seating the patient on a chair and using an adjustable strap to restrict knee extension, maintaining hip and knee angles at approximately 90° (2). It's essential to note that while these simple solutions may not guarantee high torque levels, setting the current amplitude at the maximum tolerated discomfort is likely to yield the most significant treatment effect (74). Additionally, for assessing the quality of contraction, visual inspection can be used, along with the option of manual resistance if needed. The use of visual inspection and manual resistance helps monitor progress and ensure the desired muscle response in NMES programs.

***Limitations***

We have some limitations in this review. The scope of our investigation was confined to evoked torque (non-potentiated), fatigue (torque decline), discomfort, and chronic adaptation measures (strength and muscle mass gains) in response to NMES. However, certain included studies delve into other variables that may hold relevance for future reviews, such as the force-frequency relationship, potentiated torque, M-wave, and force-time integral. Moreover, the presence of multiple outcome measures introduces complexity and makes descriptive comparisons between studies challenging. Furthermore, the considerable variability across evaluations and clinical heterogeneity among studies precluded us from conducting meta-analyses, consequently constraining this review to descriptive rather than quantitative comparisons. Lastly, despite conducting a comprehensive literature search across diverse databases, it is important to recognize that the search primarily targeted English-language journals, potentially overlooking studies in non-English publications and regional databases

The included studies exhibited scores ranging from fair to good, ranging from 4 to 7 points on the PEDro scale. An assessment combining both the PEDro and RoB-CO frameworks reveals several concerns, particularly the absence of a clear delineation of methods to eliminate carryover effects. This becomes particularly pertinent when considering outcomes linked to maximum evoked torque. While examples of insufficient randomization were infrequent, they were evident in three studies. Additionally, it is noteworthy that achieving blinding for both participants and assessors in studies involving joint angles can present inherent challenges. For forthcoming research endeavors, we advocate for participant blinding pertaining to specific outcomes that have the potential to influence performance. Furthermore, it is essential to underscore that a significant number of studies have not incorporated a preliminary familiarization session, despite its potential to impact participants' performance

In conclusion, optimal muscle length is key for maximizing torque generation during NMES. Longer muscle lengths also contribute to increased torque, while shorter lengths may result in more discomfort. Tailoring joint angles to training goals can influence contraction fatigue. Quadriceps strength gains are superior at the ideal muscle length, aiding muscle recovery. These findings are crucial for populations with limited mobility due to disuse. However, more research is needed to comprehensively assess the short- and long-term effects of varying muscle lengths on musculoskeletal adaptations after the NMES-strengthening program.

**Acknowledgments**

This study was financed in part by the Coordenação de Aperfeiçoamento de Pessoal de Nível Superior, Brazil (CAPES), Finance Code 001, Fundação de Apoio à Pesquisa do Distrito Federal (FAPDF) (grant numbers 00193-00001615/2023-00, 00193-00002357/2022-90, 00193.00001222/2021-26, 00193-00001261/2021-23), and the National Council for Scientific and Technological Development (CNPq; process number 310269/2021). M.A.V. AND J.L.Q.D are recipient of a research grant from the National Council for Scientific and Technological Development.

**Conflicts of interest**

The authors declare no conflict of interest.

**References**

1.	Herzig D, Maffiuletti NA, Eser P. The Application of Neuromuscular Electrical Stimulation Training in Various Non-neurologic Patient Populations: A Narrative Review. PM R. 2015;7(11):1167-78.

2.	Vaz MA, Baroni BM, Geremia JM, Lanferdini FJ, Mayer A, Arampatzis A, et al. Neuromuscular electrical stimulation (NMES) reduces structural and functional losses of quadriceps muscle and improves health status in patients with knee osteoarthritis. J Orthop Res. 2013;31(4):511-6.

3.	Bastos JAI, Martins WR, Junior GC, Collins DF, Durigan JLQ. Contraction fatigue, strength adaptations, and discomfort during conventional versus wide-pulse, high-frequency, neuromuscular electrical stimulation: a systematic review. Appl Physiol Nutr Metab. 2021;46(11):1314-21.

4.	Rabello R, Frohlich M, Maffiuletti NA, Vaz MA. Influence of Pulse Waveform and Frequency on Evoked Torque, Stimulation Efficiency, and Discomfort in Healthy Subjects. Am J Phys Med Rehabil. 2021;100(2):161-7.

5.	Medeiros FV, Bottaro M, Vieira A, Lucas TP, Modesto KA, Bo APL, et al. Kilohertz and Low-Frequency Electrical Stimulation With the Same Pulse Duration Have Similar Efficiency for Inducing Isometric Knee Extension Torque and Discomfort. Am J Phys Med Rehabil. 2017;96(6):388-94.

6.	Maffiuletti NA. Physiological and methodological considerations for the use of neuromuscular electrical stimulation. Eur J Appl Physiol. 2010;110(2):223-34.

7.	Modesto KAG, Bastos JAI, Vaz MA, Durigan JLQ. Effects of Kilohertz Frequency, Burst Duty Cycle, and Burst Duration on Evoked Torque, Perceived Discomfort and Muscle Fatigue: A Systematic Review. Am J Phys Med Rehabil. 2022.

8.	Vaz MA, Frasson VB. Low-Frequency Pulsed Current Versus Kilohertz-Frequency Alternating Current: A Scoping Literature Review. Arch Phys Med Rehabil. 2018;99(4):792-805.

9.	Cavalcante JGT, Marqueti RC, Geremia JM, de Sousa Neto IV, Baroni BM, Silbernagel KG, et al. The Effect of Quadriceps Muscle Length on Maximum Neuromuscular Electrical Stimulation Evoked Contraction, Muscle Architecture, and Tendon-Aponeurosis Stiffness. Front Physiol. 2021;12:633589.

10.	Silva PE, Babault N, Mazullo JB, de Oliveira TP, Lemos BL, Carvalho VO, et al. Safety and feasibility of a neuromuscular electrical stimulation chronaxie-based protocol in critical ill patients: A prospective observational study. J Crit Care. 2017;37:141-8.

11.	Allen BC, Stubbs KJ, Dixon WE. Characterization of the Time-Varying Nature of Electromechanical Delay During FES-Cycling. IEEE Trans Neural Syst Rehabil Eng. 2020;28(10):2236-45.

12.	Cavalcante JGT, de Almeida Ventura Á, de Jesus Ferreira LG, de Sousa AMM, de Sousa Neto IV, de Cássia Marqueti R, et al. Hip and Knee Joint Angles Determine Fatigue Onset during Quadriceps Neuromuscular Electrical Stimulation. Applied Bionics and Biomechanics. 2022;2022:4612867.

13.	Cavalcante JGT, Marqueti RdC, Corrigan P, de Sousa AMM, Bottaro M, Babault N, et al. The effects of knee and hip joint angles on patellar tendon loading during quadriceps neuromuscular electrical stimulation. TRANSLATIONAL SPORTS MEDICINE. 2021;4(5):587-96.

14.	Gerrits KH, Maganaris CN, Reeves ND, Sargeant AJ, Jones DA, de Haan A. Influence of knee joint angle on muscle properties of paralyzed and nonparalyzed human knee extensors. Muscle Nerve. 2005;32(1):73-80.

15.	Ventura A, Gomes L, Cavalcante J, Vieira D, Sousa CV, Marqueti RC, et al. Does Electrode Sensor Positioning over Motor Points Affect Different Portions of Quadriceps Muscle Architecture during Submaximal Evoked Torque? Journal of Sensors. 2023;2023:7.

16.	Abe T, Kumagai K, Brechue WF. Fascicle length of leg muscles is greater in sprinters than distance runners. Med Sci Sports Exerc. 2000;32(6):1125-9.

17.	Alegre LM, Ferri-Morales A, Rodriguez-Casares R, Aguado X. Effects of isometric training on the knee extensor moment-angle relationship and vastus lateralis muscle architecture. Eur J Appl Physiol. 2014;114(11):2437-46.

18.	Maeo S, Huang M, Wu Y, Sakurai H, Kusagawa Y, Sugiyama T, et al. Greater Hamstrings Muscle Hypertrophy but Similar Damage Protection after Training at Long versus Short Muscle Lengths. Med Sci Sports Exerc. 2021;53(4):825-37.

19.	Hollander K. Long-term adaptations of habitual barefoot locomotion on foot morphology and biomechanics during childhood and adolescence (PhD Academy Award). Br J Sports Med. 2019;53(9):583-4.

20.	Gordon AM, Huxley AF, Julian FJ. The variation in isometric tension with sarcomere length in vertebrate muscle fibres. J Physiol. 1966;184(1):170-92.

21.	de Sousa AMM, Cavalcante JGT, Bottaro M, Vieira DCL, Babault N, Geremia JM, et al. The Influence of Hip and Knee Joint Angles on Quadriceps Muscle-Tendon Unit Properties during Maximal Voluntary Isometric Contraction. Int J Environ Res Public Health. 2023;20(5).

22.	Fukutani A, Misaki J, Isaka T. Relationship between joint torque and muscle fascicle shortening at various joint angles and intensities in the plantar flexors. Sci Rep. 2017;7(1):290.

23.	Moo EK, Leonard TR, Herzog W. The sarcomere force-length relationship in an intact muscle-tendon unit. J Exp Biol. 2020;223(Pt 6).

24.	Hansen EA, Lee HD, Barrett K, Herzog W. The shape of the force-elbow angle relationship for maximal voluntary contractions and sub-maximal electrically induced contractions in human elbow flexors. J Biomech. 2003;36(11):1713-8.

25.	Marsh E, Sale D, McComas AJ, Quinlan J. Influence of joint position on ankle dorsiflexion in humans. J Appl Physiol Respir Environ Exerc Physiol. 1981;51(1):160-7.

26.	Fitzgerald GK, Piva SR, Irrgang JJ. A modified neuromuscular electrical stimulation protocol for quadriceps strength training following anterior cruciate ligament reconstruction. J Orthop Sports Phys Ther. 2003;33(9):492-501.

27.	Delitto A, Rose SJ, McKowen JM, Lehman RC, Thomas JA, Shively RA. Electrical stimulation versus voluntary exercise in strengthening thigh musculature after anterior cruciate ligament surgery. Phys Ther. 1988;68(5):660-3.

28.	Fahey TD, Harvey M, Schroeder RV, Ferguson F. Influence of sex differences and knee joint position on electrical stimulation-modulated strength increases. Med Sci Sports Exerc. 1985;17(1):144-7.

29.	Liberati A, Altman DG, Tetzlaff J, Mulrow C, Gotzsche PC, Ioannidis JP, et al. The PRISMA statement for reporting systematic reviews and meta-analyses of studies that evaluate health care interventions: explanation and elaboration. PLoS Med. 2009;6(7):e1000100.

30.	Moher D, Shamseer L, Clarke M, Ghersi D, Liberati A, Petticrew M, et al. Preferred reporting items for systematic review and meta-analysis protocols (PRISMA-P) 2015 statement. Syst Rev. 2015;4:1.

31.	Christie A, Snook EM, Kent-Braun JA. Systematic review and meta-analysis of skeletal muscle fatigue in old age. Med Sci Sports Exerc. 2011;43(4):568-77.

32.	Mendoza C, Kraemer P, Herrera P, Burdiles P, Sepulveda D, Nunez E, et al. [Clinical guidelines using the GRADE system (Grading of Recommendations Assessment, Development and Evaluation)]. Rev Med Chil. 2017;145(11):1463-70.

33.	Fitch S, McComas A. Influence of human muscle length on fatigue. J Physiol. 1985;362:205-13.

34.	Bremner CB, Holcomb WR, Brown CD. Knee joint angle influences neuromuscular electrical stimulation-induced torque. Athletic Training and Sports Health Care. 2015;7(4):165-72.

35.	McNeal DR, Baker LL. Effects of joint angle, electrodes and waveform on electrical stimulation of the quadriceps and hamstrings. Ann Biomed Eng. 1988;16(3):299-310.

36.	Babault N, Pousson M, Michaut A, Van Hoecke J. Effect of quadriceps femoris muscle length on neural activation during isometric and concentric contractions. J Appl Physiol (1985). 2003;94(3):983-90.

37.	Bremner CB, Holcomb WR, Brown CD, Miller MG. Assessment of Comfort During NMES-induced Quadriceps Contractions at Two Knee Joint Angles. Athletic Training &amp; Sports Health Care. 2015;7(5):181-9.

38.	Gavin JP, Cooper M, Wainwright TW. The effects of knee joint angle on neuromuscular activity during electrostimulation in healthy older adults. J Rehabil Assist Technol Eng. 2018;5:2055668318779506.

39.	Debenham MIB, Power GA. The influence of preceding activity and muscle length on voluntary and electrically evoked contractions. Appl Physiol Nutr Metab. 2019;44(3):301-8.

40.	Frigon A, Thompson CK, Johnson MD, Manuel M, Hornby TG, Heckman CJ. Extra forces evoked during electrical stimulation of the muscle or its nerve are generated and modulated by a length-dependent intrinsic property of muscle in humans and cats. J Neurosci. 2011;31(15):5579-88.

41.	Scott W, Adams C, Fisher J, Fisher S, Jones K, Mathieu B. Electrically elicited quadriceps muscle torque: Comparison at three knee angles. Physiother Theory Pract. 2019:1-7.

42.	Rassier DE. The effects of length on fatigue and twitch potentiation in human skeletal muscle. Clin Physiol. 2000;20(6):474-82.

43.	Kooistra RD, de Ruiter CJ, de Haan A. Conventionally assessed voluntary activation does not represent relative voluntary torque production. Eur J Appl Physiol. 2007;100(3):309-20.

44.	de Ruiter CJ, Hoddenbach JG, Huurnink A, de Haan A. Relative torque contribution of vastus medialis muscle at different knee angles. Acta Physiol (Oxf). 2008;194(3):223-37.

45.	de Ruiter CJ, Kooistra RD, Paalman MI, de Haan A. Initial phase of maximal voluntary and electrically stimulated knee extension torque development at different knee angles. J Appl Physiol (1985). 2004;97(5):1693-701.

46.	Kooistra RD, de Ruiter CJ, de Haan A. Muscle activation and blood flow do not explain the muscle length-dependent variation in quadriceps isometric endurance. J Appl Physiol (1985). 2005;98(3):810-6.

47.	Harnie J, Cattagni T, Cornu C, McNair P, Jubeau M. Acute effect of tendon vibration applied during isometric contraction at two knee angles on maximal knee extension force production. PLoS One. 2020;15(11):e0242324.

48.	Place N, Maffiuletti NA, Ballay Y, Lepers R. Twitch potentiation is greater after a fatiguing submaximal isometric contraction performed at short vs. long quadriceps muscle length. J Appl Physiol (1985). 2005;98(2):429-36.

49.	Foure A, Ogier AC, Guye M, Gondin J, Bendahan D. Muscle alterations induced by electrostimulation are lower at short quadriceps femoris length. Eur J Appl Physiol. 2020;120(2):325-35.

50.	Ando R, Tomita A, Watanabe K, Akima H. Knee joint angle and vasti muscle electromyograms during fatiguing contractions. Clin Physiol Funct Imaging. 2018;38(4):566-72.

51.	Lee SC, Braim A, Becker CN, Prosser LA, Tokay AM, Binder-Macleod SA. Diminished fatigue at reduced muscle length in human skeletal muscle. Muscle Nerve. 2007;36(6):789-97.

52.	Marion MS, Wexler AS, Hull ML, Binder-Macleod SA. Predicting the effect of muscle length on fatigue during electrical stimulation. Muscle Nerve. 2009;40(4):573-81.

53.	Sacco P, McIntyre DB, Jones DA. Effects of length and stimulation frequency on fatigue of the human tibialis anterior muscle. J Appl Physiol (1985). 1994;77(3):1148-54.

54.	Yanase K, Hasegawa S, Nakamura M, Tanaka H, Umehara J, Araki K, et al. Acute effect of electrical stimulation on the infraspinatus muscle using different types of muscle contractions and shoulder joint positions. Edizioni Minerva Medica. 2017;176(12):8.

55.	FRASSON VB, RASSIER DJE, HERZOG W, VAZ MA. DORSIFLEXOR AND PLANTARFLEXOR TORQUE-ANGLE AND TORQUE-VELOCITY RELATIONSHIPS OF CLASSICAL BALLET DANCERS AND VOLLEYBALL PLAYERS. Revista Brasileira de Biomecânica 2007;8:31 - 6.

56.	Noorkoiv M, Nosaka K, Blazevich AJ. Neuromuscular adaptations associated with knee joint angle-specific force change. Med Sci Sports Exerc. 2014;46(8):1525-37.

57.	Skurvydas A, Brazaitis M, Kamandulis S, Sipaviciene S. Peripheral and central fatigue after muscle-damaging exercise is muscle length dependent and inversely related. J Electromyogr Kinesiol. 2010;20(4):655-60.

58.	Kubo K, Ohgo K, Takeishi R, Yoshinaga K, Tsunoda N, Kanehisa H, et al. Effects of isometric training at different knee angles on the muscle-tendon complex in vivo. Scand J Med Sci Sports. 2006;16(3):159-67.

59.	O'Sullivan K, McAuliffe S, Deburca N. The effects of eccentric training on lower limb flexibility: a systematic review. Br J Sports Med. 2012;46(12):838-45.

60.	de Boer MD, Maganaris CN, Seynnes OR, Rennie MJ, Narici MV. Time course of muscular, neural and tendinous adaptations to 23 day unilateral lower-limb suspension in young men. J Physiol. 2007;583(Pt 3):1079-91.

61.	Visscher RMS, Rossi D, Friesenbichler B, Dohm-Acker M, Rosenheck T, Maffiuletti NA. Vastus medialis and lateralis activity during voluntary and stimulated contractions. Muscle Nerve. 2017;56(5):968-74.

62.	Maffiuletti NA, Lepers R. Quadriceps femoris torque and EMG activity in seated versus supine position. Med Sci Sports Exerc. 2003;35(9):1511-6.

63.	Bampouras TM, Reeves ND, Baltzopoulos V, Maganaris CN. The role of agonist and antagonist muscles in explaining isometric knee extension torque variation with hip joint angle. Eur J Appl Physiol. 2017;117(10):2039-45.

64.	Herzog W, Abrahamse SK, ter Keurs HE. Theoretical determination of force-length relations of intact human skeletal muscles using the cross-bridge model. Pflugers Arch. 1990;416(1-2):113-9.

65.	Hali K, Zero AM, Rice CL. Effect of ankle joint position on triceps surae contractile properties and motor unit discharge rates. Physiol Rep. 2021;8(24):e14680.

66.	Landin D, Thompson M, Reid M. Knee and Ankle Joint Angles Influence the Plantarflexion Torque of the Gastrocnemius. J Clin Med Res. 2015;7(8):602-6.

67.	Merlet AN, Cattagni T, Cornu C, Jubeau M. Effect of knee angle on neuromuscular assessment of plantar flexor muscles: A reliability study. PLoS One. 2018;13(3):e0195220.

68.	Mela P, Veltink PH, Huijing PA. The influence of stimulation frequency and ankle joint angle on the moment exerted by human dorsiflexor muscles. J Electromyogr Kinesiol. 2001;11(1):53-63.

69.	Balnave CD, Allen DG. The effect of muscle length on intracellular calcium and force in single fibres from mouse skeletal muscle. J Physiol. 1996;492 ( Pt 3)(Pt 3):705-13.

70.	Miyamoto N, Oda S. Effect of joint angle on mechanomyographic amplitude during unfused and fused tetani in the human biceps brachii muscle. Eur J Appl Physiol. 2005;95(2-3):221-8.

71.	Pincivero DM, Salfetnikov Y, Campy RM, Coelho AJ. Angle- and gender-specific quadriceps femoris muscle recruitment and knee extensor torque. J Biomech. 2004;37(11):1689-97.

72.	Akima H, Tomita A, Ando R. Effect of knee joint angle on the neuromuscular activation of the quadriceps femoris during repetitive fatiguing contractions. J Electromyogr Kinesiol. 2019;49:102356.

73.	Ichikawa K, Jiang Y, Sugi M, Togo S, Yokoi H. Joint angle based motor point tracking stimulation for surface FES: A Study on biceps brachii. Med Eng Phys. 2021;88:9-18.

74.	Conley CEW, Mattacola CG, Jochimsen KN, Dressler EV, Lattermann C, Howard JS. A Comparison of Neuromuscular Electrical Stimulation Parameters for Postoperative Quadriceps Strength in Patients After Knee Surgery: A Systematic Review. Sports Health. 2021;13(2):116-27.

75.	Toth MJ, Tourville TW, Voigt TB, Choquette RH, Anair BM, Falcone MJ, et al. Utility of Neuromuscular Electrical Stimulation to Preserve Quadriceps Muscle Fiber Size and Contractility After Anterior Cruciate Ligament Injuries and Reconstruction: A Randomized, Sham-Controlled, Blinded Trial. Am J Sports Med. 2020;48(10):2429-37.
