# Supplementary material for: Effect of muscle length on maximum evoked torque, discomfort, contraction fatigue, and strength adaptations during electrical stimulation in adult populations: A systematic review
Source: PLoS One. 2024 Jun 10;19(6):e0304205. doi: 10.1371/journal.pone.0304205 (PMC11164398; doi:10.1371/journal.pone.0304205)
Supplement: Attachment — Submitted filename: Response to Reviewers.docx [file pone.0304205.s005.docx]

_____________________________________________________________________________________

Jonathan Galvão Tenório Cavalcante MSc,

University of Brasília, Faculdade de Ceilândia

Brasília, DF, Brazil

Centro Metropolitano, Conjunto A, Lote 01

CEP: 70.840-901

Brasília, DF, Brazil

e-mail: jonathangalvao1989@gmail.com

Phone: +55 61 995128423

Fax: +55 61 31078401

April 1, 2024

Dear Professor Dr Emily Chenette

Editor-in-chief, PLOS ONE

Manuscript ID PONE-D-23-35590: Effect of muscle length on maximum evoked torque, discomfort, fatigue, and strength adaptations during electrical stimulation in adult populations: a systematic review

Thank you for the opportunity to answer the reviewers and upload our revised manuscript.

The comments from the reviewers were highly insightful and enabled us to improve the quality of our manuscript. We have made several changes to address the suggestions. A point-by-point response to the reviewers is provided bellowe, and the marked-up, and unmarked versions of the manuscript were uploaded, as well the necessary adjustments in the supplemental files.

Thank you again for your time and effort in considering this manuscript for publication.

Sincerely,

Jonathan Galvão Tenório Cavalcante

João Luiz Quaglito Durign

Laboratory of Muscle and Tendon Plasticity

Graduate Program in Rehabilitation Sciences

University of Brasília,

**Journal Requirements:**

When submitting your revision, we need you to address these additional requirements.

**1. Please ensure that your manuscript meets PLOS ONE's style requirements, including those for file naming. The PLOS ONE style templates can be found at**

**https://journals.plos.org/plosone/s/file?id=wjVg/PLOSOne_formatting_sample_main_body.pdf and**

**https://journals.plos.org/plosone/s/file?id=ba62/PLOSOne_formatting_sample_title_authors_affiliations.pdf**

**Reply:** Thank you for the review opportunity. We have implemented the requested changes as per the provided instructions.

**2. Note from Emily Chenette, Editor in Chief of PLOS ONE, and Iain Hrynaszkiewicz, Director of Open Research Solutions at PLOS: Did you know that depositing data in a repository is associated with up to a 25% citation advantage (https://doi.org/10.1371/journal.pone.0230416)? If you’ve not already done so, consider depositing your raw data in a repository to ensure your work is read, appreciated and cited by the largest possible audience. You’ll also earn an Accessible Data icon on your published paper if you deposit your data in any participating repository (https://plos.org/open-science/open-data/#accessible-data).**

**Reply:** We appreciate your valuable suggestion; however, at the moment, we are not interested in pursuing the proposal. All data will be made available upon request to the corresponding author. Thank you for your understanding.

**3. "We note that the grant information you provided in the ‘Funding Information’ and ‘Financial Disclosure’ sections do not match. When you resubmit, please ensure that you provide the correct grant numbers for the awards you received for your study in the ‘Funding Information’ section.**

**Reply:** Thank you for identifying this mistake. We will make the adjustment in the resubmission process.

**4. Thank you for stating in your Funding Statement:**

**" This study was financed in part by the Coordenação de Aperfeiçoamento de Pessoal de Nível Superior, Brazil (CAPES), Finance Code 001, Fundação de Apoio à Pesquisa do Distrito Federal (FAPDF) (grant numbers 00193-00001615/2023-00, 00193-00002357/2022-90, 00193.00001222/2021-26, 00193-00001261/2021-23), and the National Council for Scientific and Technological Development (CNPq; process number 310269/2021). M.A.V. AND J.L.Q.D are recipient of a research grant from the National Council for Scientific and Technological Development."**

**Please provide an amended statement that declares *all* the funding or sources of support (whether external or internal to your organization) received during this study, as detailed online in our guide for authors at http://journals.plos.org/plosone/s/submit-now. Please also include the statement “There was no additional external funding received for this study.” in your updated Funding Statement.**

**Please include your amended Funding Statement within your cover letter. We will change the online submission form on your behalf.**

**Reply**: Thank you for the observations. We have incorporated the requested changes in the cover letter (while maintaining all the previous information), as follows: *“This study was financed in part by the Fundação de Apoio à Pesquisa do Distrito Federal (FAPDF), https://www.fap.df.gov.br/ (grant numbers: author JGTC: 00193-00001615/2023-00, author JLQD: 00193-00002357/2022-90, author JLQD: 00193.00001222/2021-26, author JLQD: 00193-00001261/2021-23), and the National Council for Scientific and Technological Development, https://www.gov.br/cnpq/pt-br (CNPq; process number 310269/2021; 402816/2023-4, author JLQD).* *There was no additional external funding received for this study.”*

**4. Please include captions for your Supporting Information files at the end of your manuscript, and update any in-text citations to match accordingly. Please see our Supporting Information guidelines for more information: http://journals.plos.org/plosone/s/supporting-information.**

**Reply:** We apologize for the oversight. Our Supporting Information files are named as *Supplemental Digital Content* (SDC). We have added the captions for each of our three SDC at the end of our manuscript, and we have adjusted in-text citations as requested. Moreover, the SDC 1 was updated to match the changing in the main text pages; the SDC 2 was corrected because its title was incorrectly written as “Supplemental Ditigal Content 1”; and the SDC 3 was updated because a reference (Kooistra et al., 2005) had its assessment corrected.

**6. Please review your reference list to ensure that it is complete and correct. If you have cited papers that have been retracted, please include the rationale for doing so in the manuscript text, or remove these references and replace them with relevant current references. Any changes to the reference list should be mentioned in the rebuttal letter that accompanies your revised manuscript. If you need to cite a retracted article, indicate the article’s retracted status in the References list and also include a citation and full reference for the retraction notice.**

**Reply:** Thank you for directing our attention to an overview of our reference list. There is no retracted study included. After considering the Reviewer’s comments, we have added some studies, corrected the citations, and removed an article that was incorrectly cited (*Hollander K. Long-term adaptations of habitual barefoot locomotion on foot morphology and biomechanics during childhood and adolescence (PhD Academy Award). Br J Sports Med. 2019;53(9):583-4*.)

**REVIEWER 1**

Dear Reviewer, thank you for your valuable comments. We have tried to address them to the best of our capabilities, and we believe that the manuscript has significantly improved based on your suggestions. Your comments have been addressed point-by-point and are provided below.

**Reviewer #1: This systematic review paper analyses the effect of muscle length on parameters such as evoked torque, fatigue response/fatigability, discomfort, and strength and muscle size changes in studies using neuromuscular electrical stimulation. Systematic analyses were done for all the abovementioned parameters, while only evoked torque was eligible for a meta-analysis due to the heterogenicity and scarcity of studies on the other parameters. The review shows that there is an optimal muscle length at which the highest torque can be evoked. Conversely, shorter and longer muscle lengths provide lower torque levels, much in line with previous studies of the muscle force-length relationship. The observed fatigue appears to follow the highest torque production, so that the muscle length (joint angle) that provides the highest torque also gives the most pronounced fatigue response. For discomfort, there is no clear pattern, although some evidence suggests that electrically evoked contractions at shorter muscle lengths may be less comfortable. Only one study investigated the effect of muscle length on training adaptations, showing that training with knee flexion was superior to an extended knee for strength adaptations. The review concludes that training interventions should aim to use the optimal muscle length as opposed to short muscle lengths, unless specific factors suggested otherwise. Meanwhile, training with longer muscle lengths may also be effective for stimulating muscle hypertrophy, but more evidence is needed to draw firm conclusions.**

**General comments: There are no line numbers for the first half of the manuscript. The line numbers start in the results section. I have deleted the present line numbers and inserted them again starting from the title page (title starting in line 1) and onwards. The main body of evidence in this manuscript revolves around the investigation of evoked torque at different muscle lengths / joint angles. The conclusion very closely follows knowledge from the force-length relationship. Please acknowledge that this knowledge would be expected, although some practical aspects of testing muscle length in vivo might have had an influence on results from human muscle. For the other parameters, meta-analyses were precluded. Please be very specific on what issues need to be addressed in future studies in your discussion section, and also be specific with scenarios/patient groups where NMES provide rehabilitation opportunities that cannot be replaced by other rehabilitation strategies. This would help clinicians and future studies. Even though not explicitly stated in title, abstract or inclusion criteria, all studies included use isometric contractions to compare muscle lengths. Please make sure to clarify that only isometric contractions were used for analyses. Otherwise, studies such as PMID: 31423755 could be included for the analysis of training outcomes.**

**Reply**: We are thankful for the opportunity to learn from the reviewer’s perspectives, through pointing out the newly added literature, and by explaining or correcting our manuscript information. The reviewer raised limitations we had not mentioned. We have carefully reviewed each comment and made the appropriate adjustments/explanations. I hope the reviewer will find the work we have done acceptable.

Specific comments

**L91-93: This statement about optimization of NMES sounds like it’s mostly related to the analyses of training outcomes and discomfort, which are likely the most clinically relevant analyses, but which are also the areas with the least/weakest data. Please rephrase and make sure the statement also relates well to the analysis of evoked torque and fatigue.**

**Reply**: Thank you for directing our attention to this necessary adjustment to add clarity. We have made some modifications throughout the first paragraph to comply with your recommendation, as follows: lines 88 – 100): These factors should interact to produce greater evoked ~~contractions with minor discomfort to obtain maximum NMES-efficiency~~ force, and speed up strengthening/hypertrophy while attenuating the perceived discomfort and contraction fatigability. Accordingly, muscle length is a ~~specially~~ widely recognized modulator of the force generation capacity of muscle fibers and the stimulus for strengthening and hypertrophy when it comes to voluntary exercise (18-20). As such, the optimal muscle length (or ~~optimal~~ ideal, or preferable) for NMES, would be at the plateau region of the force-length relationship, obtained by manipulating joint angle (21-23). However, to date, there are~~is~~ no summaries~~y~~ of the scientific data on how the NMES effects can be optimized by the “best joint angle”, which may limit ~~the benefits~~ standardization of NMES-based programs and novel approaches to ~~mitigate perceived discomfort and fatigue.~~ improve performance during NMES delivery, in addition to the long-term results.

**L94-107: This section currently lacks a concluding remark or point.**

**Reply**: Thank you for your comment, we have made several changes in the second paragraph, as follows (Lines 101-122): The muscle force-length relationship of single muscle fibers *in vitro* is classically known to display an ascending~~ant~~ and a descending limb, with a plateau in between, where the maximal~~um~~ tension is achieved (21), ~~and has been further~~ also supported by recent research (24). ~~Moreover,~~ *~~in~~ In vivo,* ~~experiments must account for the moment arm for each muscle because~~ the joint angle-torque relationship becomes the practical translation of the force-length relationship for assessing human performance, taking into account that different moment arms, muscle-tendon unit architecture, and joint characteristics yield different shapes of the torque curve as the joint angle changes (11, 15, 22, 25). As such, ~~The~~ the early study ~~of~~ by Marsh et al. (26) brings relevant data showing the different shapes of the torque-angle relationship of the dorsiflexors according to the type of activation (maximal voluntary contraction [MVC] or stimulated at different frequencies). However, while the dorsiflexors may show progressively greater evoked torque as the ankle angle ~~is more~~moves towards plantarflexion (26), for the quadriceps muscle, the torque is usually greater closer to 60º of knee flexion ~~considering a range from 30º to 90º~~ (16), and for the biceps brachialis, the torque is usually greater close to 90º of elbow flexion (25). ~~These divergent findings can likely be attributed to a multitude of factors, including differences in moment arms, the overall joint range of motion, muscle architecture, and the characteristics of the in-series elastic tendinous tissues (11, 15, 22).~~ These discrepancies in the torque-angle relationship for different joints reveal that this branch of knowledge must be broadened (for more joint angles and joint types) and systematically compiled so that researchers and clinicians can make evidence-based decisions when prescribing NMES.

**L109: Is this narrative review the only instance where short muscle lengths are mentioned as being less comfortable, and without a reference to an original study?**

**Reply**: We have added a reference (PMID: 8434879) that directly shows that contractions exclusively at short muscle lengths cause muscle cramps, while muscle lengthening can actually interrupt the cramp. Moreover, several adjustments have been made in the third paragraph, as follows (Lines 123-135): When applying NMES, it is essential not only to consider torque generation but also to minimize the level of perceived discomfort~~. A narrative review (1) informed that a shortened position~~ Forceful contractions at short muscle lengths (e.g., extended knee for knee extensor stimulation) can cause painful muscle cramps (1, 27)~~could lead to cramp-like contractions~~. Therefore, during the application of NMES, in addition to biomechanical considerations, alterations in muscle length may contribute to varying levels of perceived discomfort. ~~Conversely~~ Thus, whenever possible, it is recommended ~~that~~ that the NMES training be started at the 'ideal' muscle length (i.e., ~~at~~ the joint angle that allows the greatest evoked torque generation) and that it subsequently progresses ~~towards a~~ to isometric evoked contractions at longer muscle lengths. Despite the absence of evidence indicating that this approach diminishes discomfort during NMES sessions, it may ~~serve to~~ mitigate the heightened risk of early muscle damage associated with training at longer muscle lengths (6, 28).

**L115: What is meant by “progress towards longer muscle length” in this context?**

**Reply**: This phrase means isometric contractions at longer muscle lengths. We have adjusted the text to make it clearer (Lines 129-132): *“*Thus, whenever possible, it is recommended ~~that~~ that the NMES training be started at the 'ideal' muscle length (i.e., ~~at~~ the joint angle that allows the greatest evoked torque generation) and that it subsequently progresses ~~towards a~~ to isometric evoked contractions at longer muscle lengths*”.* Moreover, several changes were necessary in the third paragraph, as already demonstrated in the commentary above.

**L127: Throughout the article, please be very clear on what is meant by “fatigue” and “fatigability”, which is not the same thing and may have different definitions in different articles. It would be helpful if you also specified the fatigue/fatigability definitions used by your analyzed studies.**

**Reply**: Thank you for this important comment. Both these declines in contraction force induced by NMES are types of performance fatigability (i.e., decline in an objective measure of performance over a discrete period of time; Enoka and Duchateau, 2016), that hereafter in this review is referred to as “contraction fatigability” or simply “fatigability”.” In the revised manuscript we have adopted the taxonomy of Enoka and Duchateau, as recommended (Enoka RM and Duchateau J. Med Sci Sports Exerc 2016;48(11):2228-2238). We now describe the decline in force induced by NMES as a type of “performance fatigability” and subsequently use the term “fatigability” and occasionally “contraction fatigability” throughout the manuscript.

**L131: This statement covers the importance of the analyses of training outcomes. See my first specific comment and make sure the statements cover all your outcomes fairly.**

**Reply**: Thank you for the comment, we have made several changes in the fourth paragraph. We believe these changes effectively address the points you raised, and we sincerely hope that our revisions align with your expectations (Line 136 – 160): A previous study found that NMES-induced quadriceps fatigability, i.e., the reduction in force generating capacity (29), depends on muscle length changes due to hip and knee joint angles (14), where fatigue is greater at a knee flexion of 60° compared to 20°. In the case of a 60° knee flexion, a supine position leads to earlier fatigue, which means greater performance fatigability (30), while with a 20° knee flexion, a supine position delays fatigue (here defined as a decline in an objective measure of performance over a discrete period of time) (11, 30). It has also been claimed that NMES at a shortened position (full knee extension) for strengthening the quadriceps promotes greater joint soft tissue protection after anterior cruciate ligament repair (31). However, according to the authors, the strength gains observed were lower than expected, considering previous studies that used a knee flexion angle of 60º (31, 32). These results corroborate those of a ~~previous~~ pioneering study (33) that reported a greater strengthening effect for an elongated position (65º of knee flexion) compared to a shortened position (~~15º of~~ full knee extension ~~flexion~~). ~~(24). A previous study found that NMES-induced quadriceps fatigue, depends on muscle length changes due to hip and knee joint angles (14). Fatigability is greater at a knee flexion of 60° compared to 20°. In the case of a 60° knee flexion, a supine position leads to earlier fatigue, while with a 20° knee flexion, a supine position delays fatigue compared to a seated position (11).~~ While abundant and emerging data exist~~s~~ ~~on~~ to address the influence of muscle length on the benefits of NMES training programs,~~-evoked force, there has not been a systematic compilation in a review to identify the optimal muscle length for enhancing muscle adaptations and reducing NMES-induced contraction fatigue and perceived discomfort~~ to date, no systematic reviews have identified the optimal muscle lengths for the generation of higher evoked torque with lower discomfort and contraction fatigability, and a greater strengthening/hypertrophy effect during NMES rehabilitation and training programs.

**L172: Not all readers will be aware of what ‘grey literature’ means. Please specify.**

**Reply**: We have made the adjustment as follows (Lines 202-205): The grey literature (available literature not published under a rigorous, peer-reviewed, independent scientific review system) was not searched, in order to avoid introducing bias and low-quality designs that could reduce the validity of our results.

**L176-182: As mentioned in the general comments, all studies seem to use isometric contractions to compare muscle lengths on the different parameters. Please make sure to specify the reason either in your inclusion criteria or otherwise. Again, I don’t see why e.g. PMID: 31423755 does not live up to the inclusion criteria.**

**Reply**: Thank you for raising the concern regarding the type of contraction and for highlighting this relevant study. Initially, we had two expectations in mind based on our previous studies: (1) that none or few studies would have compared a restricted range of motion during dynamic NMES training; (2) acknowledging that, even if such a study existed, finding another one similar enough to allow for a meta-analysis would be challenging. Consequently, we chose not to restrict the type of contraction in the search strategy or inclusion/exclusion criteria to avoid potentially overlooking relevant clinical information from studies with different types of contractions. Unfortunately, we did not identify any NMES studies with dynamic contractions comparing muscle lengths. Additionally, the study you mentioned (PMID: 31423755) is on our list of studies excluded after abstract screening. Specifically, it was excluded because there is no angle comparison during/after NMES training, as the authors explicitly stated: “*electrically evoked contractions at near-optimal length only*.” Furthermore, their training protocol involved volitional contractions, so the study could not be included, as it would introduce confounding effects related to joint angle. However, we sincerely appreciate and thank the reviewer for bringing up the relevant study, which we have incorporated in the reference list as solid evidence to strengthen our discussion.

We acknowledge and apologize for any lack of clarity in our inclusion criteria. As per criteria ‘ii’ and ‘iii,’ our interest lies in, respectively, "*electrically induced muscle contraction as an intervention to generate maximum torque, contraction fatigability, perceived discomfort, or strength training adaptations*" and "*comparisons of different muscle lengths (i.e., joint angles)*." To better reproduce this, we have adapted the main text as follows (Lines 210-215): “ii) comparisons of different muscle lengths (i.e., joint angles) during or after electrically induced muscle contraction as an intervention to generate maximum torque, contraction fatigability, perceived discomfort, or strength training adaptations; ~~iv) reported results that could be converted to a standardized effect (36); v)~~ iii) healthy or clinical adult populations (≥ 18 years old). Studies were excluded if they investigated the vocalization, respiratory, or pelvic floor muscles.”

**L198: Do you think that the optimal length for torque production during twitch, doublet, or continuous stimulation is the same? Please comment**

**Reply**: We greatly appreciate this important comment. Doublets, triplets, or octets have been employed to elicit a more robust force response and are considered less susceptible to the influence of previous stimuli (Ruiter et al., 2008). Given our specific interest in maximal evoked contractions under fresh conditions, we reasoned that even twitch data would reliably reflect only length changes. Therefore, we opted to maintain the inclusion of these alternative methodologies. Furthermore, despite ongoing debates and conflicting results in the literature, no prior meta-analyses have compared different NMES techniques. Recognizing the significance of this gap, we have addressed this aspect in the limitations section of our study (Lines 771-774).

**L202: Replace “where” with “was” or “were”.**

**Reply**: We apologize for the mistake and thank the Reviewer for kindly pointing out the appropriate correction. We have corrected this to “were”. Moreover, we have reduced the text length (Line 235 – 237): *“*When ~~the data where were not available in tables or in the results section, we extracted from the graphs~~ the study results were only available in graphs, we extracted the data~~m~~ using ~~the~~ ImageJ software

**L217: Make sub header “Quality of Evidence” italic/cursive**

**Reply**: We apologize for the mistake and thank the Reviewer for kindly pointing out the appropriate correction. We have adjusted this accordingly.

**L253: Some studies find ~70 degrees knee flexion to be optimal for MVC torque production, e.g. DOI: 10.1016/j.jbiomech.2004.02.005. Please acknowledge that the range for optimal length may be wider than you have currently written. Also, ref 33 does not include quadriceps strength measurements, but dorsiflexors, as I understand it.**

**Reply:** This comment addresses a crucial consideration that we had previously deliberated upon. Following the reviewer's feedback, we conducted an additional search to identify studies that could substantiate a broader range for the optimal knee angle. Initially, we had selected the optimal range as being between 55º and 65º. However, in contemplating an extension to 70º, we also questioned the possibility of broadening the range to 50º. Based on a newly included reference (Šarabon et al., 2021), we successfully expanded the optimal (or midrange) angle to 50-70º. The study referenced by the reviewer (Pincivero et al., 2004) also played a pivotal role in our decision-making process. Given that the studies included in our analysis encompassed a mixed population of females and males, combining 50-70º as the optimal muscle length seemed more reasonable than combining 70-90º as a long muscle length. This rationale is particularly supported by the MVC values reported by Pincivero et al., (2004) (combined mean of males and females: n = 29, 50º: 199.44 ± 58.86, 70º: 220.20 ± 78.34, 90º: 168.28 ± 76.54; see response to **L339-341**). It is important to emphasize that the adjustment in the optimal angle range is a strategic move aimed at enhancing compatibility for meta-analysis. As more studies become available in the future, we anticipate that the meta-analyses could become more specific (e.g., 60º vs. 70º). Notably, this reclassification did not necessitate the removal of any studies from the meta-analysis. On the contrary, it enabled the inclusion of Skuvirdas et al. (2010) in a new meta-analysis, a study that was previously not added to the meta-analysis due to a lack of a corresponding study for knee joint comparisons (50º vs. 90º), as elucidated in the discussion related to **L499**. However, it is essential to mention that data from Guerrits et al. (2005) required updating, as detailed in the response to **L339-341.** Regarding reference 33, it was mistakenly cited; Rassier was the correct reference. We have made the necessary adjustment in the new version of the manuscript.

**L295: Correct “3” to “three”**

**Reply**: We apologize for the mistake and thank the Reviewer for kindly pointing out the appropriate correction. We have adjusted this accordingly.

**L312: Please elaborate briefly on what ‘extra forces’ means, as some readers may not be familiar with the manuscript by Frigon et al.**

**Reply**: You are correct. As requested, we have added this explanation (Lines 350-354): … and the extra forces with preceding activity, i.e., NMES-induced contractions through central pathways that lead to an increase in force increments that is disproportionate to the input/electrical current applied (51). In this case, a 25 Hz-10 0Hz-25 Hz stimulation was applied at different ankle joints and the ratio of the torque generated in the second over the first 25 Hz stimuli was calculated.

**L321: Please briefly describe what a Russian current is.**

***Reply****:* ~~Notably, one study~~ The exceptions were the two studies by Bremner et al. (46, 48) that used a median frequency current (2500 Hz delivered in bursts of 50 Hz, also known as Russian current). ~~deviated from the use of pulsed current and opted for the utilization of the Russian current.~~

**L328: There are some inconsistencies regarding the use of numerals or numbers written with words. Please keep consistent. In this case change for ”23”, probably**

**Reply**: We apologize for the mistake and thank the Reviewer for kindly pointing out the appropriate correction. We have adjusted “twenty-three” to “23”.

**L339-341: How were the measurements combined? As an average for the angles in each category? Please comment.**

**Reply**: This is a very important question and we have improved the description in lines 383-390. Data were combined using the Cochrane recommendation (<https://training.cochrane.org/handbook/archive/v6.3/chapter-06>). The calculation itself was performed by inserting the variables (n, mean, SD) here: <https://www.statstodo.com/CombineMeansSDs.php>. After revising the averages for the angles in each category, we identified two adjustments that had to be performed during this review. Firstly, there is a subtle detail in the study by Guerrits et al. (2008) that we had overlooked. In the methods section, the authors reported having eight healthy participants. However, in Figure 1-A, there are triplet torque data for five healthy participants, and in Figure 2-B, there are triplet torque data for seven healthy participants. Initially, we considered their sample size as eight participants in the meta-analysis and extracted data from Figure 1-A. However, it turns out that this figure represented data from a pilot experiment, and we have now extracted data from Figure 2-A. Secondly, for the n = 7 of Guerrits et al. (2005), when two means are combined, the sample size is actually 14, and when three means are combined, the sample size is 21. These adjustments have been made in the reformulated meta-analyses. Fortunately, this did not change the significance of any meta-analysis. Below are the values for Guerrits et al. (2005) as they were extracted from the Figure 2-A, along with an example of combining the torque for 50º, 60º, and 70º into a single value:

| Supramaximal Triplet stimulation (Nm) | | | | | | |
| --- | --- | --- | --- | --- | --- | --- |
| 30º | 40º | 50º | 60º | 70º | 80º | 90º |
| 78.63 ± 13.04 | 92.90 ± 12.72 | 101.52 ± 16.37 | 106.33 ± 12.75 | 104.26 ± 13.83 | 101.42 ± 13.38 | 100.97 ± 18.25 |
|  |  | Combined: 104.03 ± 13.80 | | | Combined: 101.19 ± 15.37 | |
| N = 7 | N = 7 | N = 21 | | | N = 14 | |
| Very Short | Short | Optimal | | | Long | |

**L372: If this is only true in the 4 out of 6 cases, it should be specified that the remaining studies did not reach the same conclusion.**

**Reply**: We have rephrased the text to better reflect our observation (Lines 426-439)*:* More highly flexed knee joint angles were more fatiguing for the quadriceps ~~for~~ in five out of the six studies: 20º vs. 60º (14), 50º vs. 100º (28), 60º vs. 110º (59), ~~and~~ 15º vs. 90º (60), and 30º vs 60º vs 90º (40), ~~However, in cases where the comparison involved more than two joint angles, the initial capacity for torque development played a significant role in influencing the results regarding fatigability. In~~ while in the investigation by Marion et al. (61), contraction fatigue was notably pronounced at the joint angle that exhibited the highest pre-fatigue force~~, while conversely,~~ and the least contraction fatigue was observed at the joint angle characterized by a lower pre-fatigue force. In this instance, 65º of knee flexion demonstrated a greater pre-fatigue force compared to angles of 20º and 90º. ~~The study conducted by~~ However, Rassier (40) exhibited contradictory outcomes and reporting. The authors~~y~~ indicated a more substantial relative decline in torque at the most elongated position (90º compared to 60º and 30º of knee flexion), ~~while~~ but the textual content consistently referred to greater contraction fatigue occurring at the most shortened muscle length.

**L372-375: Do you think that the for the fatigability, the muscle length itself plays a role, or do you think that it is simply the resulting force/torque that decides the fatigue development? Perhaps something you could discuss in your discussion section.**

**Reply:** This is an intriguing question. We have done our best to address this question in two separate paragraphs from Lines 652 to 688 (***Contraction fatigability*** section).

**L418-425: The only study investigating the effect of training at various muscle lengths only use a (seemingly) very short quadriceps length and a flexed position. As the short length seems to be very short,**

**Reply**: It seems that a request from the Reviewer was accidently deleted from the reviewer’s comment. Thus we have tried to anticipate the point made and included some concerns about limitations in the discussion (‘*Strengthening by NMES training’* section) regarding knee and hip angles. Moreover, as requested for **L611-612**, we have added quantitative information in the results section (Lines 476-487).

**L430-432: The fact that the optimal muscle length yields greater contraction force than short or long muscle lengths is rather self-explanatory. Please rephrase. Perhaps that the force-length relationship is apparent in electrically evoked contractions in the quadriceps femoris in vivo.**

**Reply**: Thank you for noticing this. We have rephrased this part as follows (Lines 484-488): We found that, for the quadriceps femoris muscle, the optimal muscle length for ~~greater~~ evoked torque was obtained ~~in the~~ at ~~optimal muscle length~~ midrange (50º-70º) compared to short (regardless of stimulation method) and long muscle lengths (during nerve stimulation), reflecting its force-length relationship.

**L433: Long lengths allowed greater torque than and very short lengths as I understand it.**

**Reply**: Thank you for noticing this. We have also added the very short length (Lines 497).

**L437: replace “plantarflexors” with “plantar flexors”**

**Reply:** We apologize for the mistake and extend our gratitude to the reviewer for kindly pointing out the necessary correction. We have made the required adjustments, as requested (Line 503).

**L447-450: Here you are very conservative when discussing the potential usefulness of training at long muscle lengths. It would be better if you could be clear on the potential benefits/drawbacks of training at long muscle lengths and what still needs to be investigated to make more firm conclusions.**

**Reply**: Thank you for your insightful comments. We have made several changes in the second paragraph of the discussion (Lines 508-526): When considering a given current amplitude, a shorter ~~length of the~~ quadriceps muscle length seems to ~~result in~~ induce increased discomfort compared to the neutral position, although no definitive conclusions can be drawn regarding other muscle lengths and different muscles. NMES-induced contraction fatigue appears to be greater when the muscle length enables greater torque generation in the pre-fatigue condition. ~~While~~ A protocol~~s~~ conducted at the ideal muscle length yielded greater quadriceps strength gains compared to a very short length, probably due to the greater mechanical work performed by the muscle at optimal length. ~~s, there is limited data available for comparing other muscle lengths and muscles~~. Taken together, ~~These t~~hese findings suggest that prioritizing the optimal muscle length should be the initial choice for NMES interventions, avoiding shortened, low-demanding muscle lengths if clinically viable. However, indirect evidence from the included studies (63, 65, 66) and voluntary training elsewhere (67, 68) point to the use of long quadriceps muscle lengths ~~while the use of long or short muscle lengths should be carefully considered and selected based on diverse clinical factors.~~ as a means of increasing mechanical stress during contraction and likely improving muscle force/mass and tendon properties, while the hamstrings may still benefit from training at shorter lengths (69). Therefore, there are still many questions to be answered regarding NMES effects according to muscle length, with further research needed.

**L454-458: I believe the finding of the capacity of the quadriceps to produces more torque at optimal muscle lengths is not quite as novel as it is made out to sound here.**

**Reply**: We agree and have rephrased the sentence (Lines 518-521): While the force-length relationship is a well-established property of muscles fibers (21), this is the first meta-analysis ~~marks the first instance of~~ confirming force disparities across multiple quadriceps femoris muscle lengths during electrical stimulation.

**L463-466: Should this part about short vs. long muscle lengths not be in the following section instead of in the section about short vs. optimal muscle lengths? Depending on how you define “long” of course, and whether you want to call 86.9 degrees long or optimal.**

**Reply**: Thank you to the reviewer for this relevant suggestion. We also considered it more appropriate to move this part to the following section on *'Very short'* and *'short'* vs. *'long' quadriceps femoris muscle length (*Lines 544-559*)*.

**L476-477: This sentence end abruptly. Aligning with long-term goals of what?**

**Reply**: We have deleted the vague conclusion because the benefits (or long term goals) have been previously stated as muscle and tendon improvements (Lines 538-544): (21). ~~Indeed, it is possible to obtain greater torque at 50º of knee flexion, compared to 90º during quadriceps muscle belly stimulation (43).~~ However, it is important to consider that long muscle lengths tend~~s~~ to induce greater tensile stress (68), a factor favorable to enhance~~d~~ muscle (serial and parallel sarcomere) growth (67) and improve~~d~~ tendon mechanical properties ~~of tendons~~ (68) ~~(59)~~. This, in turn, lends support to the idea of ~~favoring~~ choosing longer muscle lengths to accelerate the recovery of muscle mass. ~~for prolonged utilization, aligning with long-term goals.~~

**L499: Please also explain why reference 57 could not be a part of a meta-analysis.**

**Reply**: Previously, this reference could not be meta-analyzed because there was no matching study that compared optimal vs. long muscle lengths during muscle belly/motor point stimulation. However, after reclassifying quadriceps muscle length according to knee angle, it became possible to include it in a meta-analysis.

**L538: This sentence is not clear. Did Marsh et al. state that they didn’t reach MVC torque levels because of a lack of synergist activation during evoked contractions? And what are the implications for the conclusions of this section if they misinterpreted their findings in such a way?**

**Reply**: I appreciate your recognition of this unclear sentence. We have deleted it without compromising the relevant aspects of Marsh et al. (Lines 620-635): Mela et al. (38) stimulated the dorsiflexors through the stimulation of the deep peroneal nerve, while Marsh et al. (26) applied the stimulation directly over the tibialis anterior muscle belly. Specifically for Marsh et al. (26), who~~ich~~ assessed several joint angles (from 30° of plantar flexion to 20º of dorsiflexion in steps of 5°), the greatest torque was obtained at 10º of plantar flexion. ~~Marsh et al. (26) may have had a misconception regarding the argument that the evoked torque didn't reach MVC values due to their stimulation method not including other dorsiflexors.~~ In the Mela et al. (38) study, nerve stimulation applied to activate all four dorsiflexors ~~still~~ resulted in an average evoked torque of 50% of MVC regardless of ankle joint angle~~in the respective angle position~~. In healthy volunteers, evoked torque rarely reaches 100% of MVC (1). Both authors, despite using different~~ing~~ stimulation methods, consistently found that torque generation was greater in the plantar flexed (lengthened) position compared to the dorsiflexed (shortened) position, which also agrees whti the force-length relationship of the dorsiflexors, whose plateau occurs at 30º of plantar flexion (64). This agreement ~~underscores~~ emphasizes the influence of joint angle (and therefor muscle length) on torque production, irrespective of the specific details of the stimulation method~~'s specific details~~.

**L553: What is meant by ”submaximal curves” in this context?**

**Reply**: We have deleted several lines related to this information because it was indeed out of context (Lines 641-646): Future studies could ~~explore~~ target~~ing~~ the peak region of the force-angle relationship with NMES to more efficiently enhance elbow flexor strength ~~more efficiently~~. ~~Utilizing submaximal curves from evoked contractions for clinical interventions is plausible given that most daily activities did not require maximal effort. Investigating this approach could optimize NMES protocols for more efficient elbow flexor strength improvement~~.

**L574-577: Please acknowledge that studies still need to confirm that training at optimal muscle length and higher torque production leads to greater improvements in strength and/or muscle hypertrophy compared to lower torques/suboptimal muscle lengths.**

**Reply***:* We agree and have softened our assumptions based on the need for further studies, as follows (Lines 653-671): Therefore, the higher contraction fatigue observed at optimal length should not preclude its preferential utilization in NMES strengthening programs, as it may still maintain the desired high muscle-tendon load (11, 15). However, new studies are needed to explore long-term outcomes. Additionally, for muscles spanning multiple joints, such as the quadriceps femoris, an added stretch, through hip extension, may also accelerate the onset of contraction fatigue and modulate strengthening adaptations (14), so this biomechanical aspect should not be overlooked when positioning the body parts. ~~While At last~~Finally, while the expectation that shorter muscle length generates less force, leading to reduced contraction fatigue compared to the optimal length, holds true for the quadriceps femoris, the same principle does not uniformly apply to the tibialis anterior muscle (62)~~(42)~~, opening the field for new studies to explore how different muscles respond acutely according to their fiber length and mechanical properties during NMES. ~~Assuming that increased mechanical loading at the muscle-tendon unit potentially leads to greater and possibly faster muscle adaptation the higher fatigue observed at optimal length length~~ ~~serves as evidence of the increased work induced by NMES~~. ~~This underscores the importance of considering optimal length over shorter muscle lengths in long-term clinical trials~~. ~~Additionally, for muscles spanning multiple joints, such as the quadriceps femoris, an added stretch, like hip extension, may accelerate the onset of fatigue and intensify its effects (12).~~

**L574-581: You cannot equate accelerated fatigue and an increased muscle hypertrophic response without a reference underpinning this statement. Muscle work can be very fatiguing without leading to muscle hypertrophy.**

**Reply**: We agree and have decided to omit this statement, opting for a more general comment, as demonstrated in the response to **L574-577**.

**L599-600: By “it lacks”, do you mean the study of Yanase et al., or the method that they used?**

**Reply***:* We have adjusted the statement to add clarity (Lines 689-691): Finally, the study by Yanase et al. (63) ~~present a limitation as it~~ ~~lacks~~ did not include a means of ~~torque~~ monitoring torque, thereby restricting the comprehensive interpretation of the reported~~ir~~ discomfort data.

**L611-612: As you only have one study in this analysis, please be more specific and include the increase in strength using numbers or %, and with some statistical indication of certainty, such as 95 % confidence intervals or similar, as provided in the reference. This information could also be inserted in the results section instead.**

**Reply**: The requested information has been provided in the results section. Furthermore, we have enhanced the details in the respective paragraph in the discussion (Lines 698-742).

**L613: ”also” is redundant.**

**Reply**: We thank the Reviewer for kindly pointing out the appropriate correction. We have adjusted the information in lines 712-713: ~~because no~~ No ~~more~~ further studies~~y~~ are~~i~~s available for evaluating longer muscle lengths and ~~also for~~ hypertrophy.

**L616-617: Please elaborate on how they reach this conclusion and how it aligns with the results of the present study.**

**Reply**: Adjusted as follows (Lines 732 – 739): Despite the limited number of studies, a recent review (85) on the effects of NMES on ~~the~~ knee post-surgery rehabilitation ~~considers the knee angle a critical parameter that may impact functional goals. Moreover, they conclude that a 60º angle or closer would be preferable if it is clinically safe~~ recommended positioning the patient at a 60º knee flexion angle if medically safe, justifying this advice by the anticipated greater torque in this position. Additional research on the adaptation of strengthening through NMES training with different joint positions is important to gain better understanding of its implementation in clinical settings.

**L624: It is not clear from the description how the leg is secured and positioned. Please elaborate**

**Reply***:* We have rephrased this information (Lines 725-729): “*In resource-constrained clinical settings, particularly for bed-bound patients, a practical approach to managing joint angles during isometric contractions is ~~using knee support with an ankle weight~~ by obtaining the desired knee flexion angle using a support under the lower limb (e.g., triangular wedge pillow) and ankle weights/restraints.”*

**L630: Are you saying that the current amplitude matters more than the precise muscle length?**

**Reply**: We apologize for the lack of clarity. We have made adjustments to the text in an attempt to better convey the importance of being guided by perceived discomfort: It is~~'s~~ essential to note that ~~while these simple solutions may not guarantee~~ to obtain high torque levels and, consequently, the most significant effect with these simple solutions, ~~setting~~ the current amplitude must be increased up to ~~at~~ the maximum tolerated discomfort (85) ~~is likely to yield the most significant treatment effect~~ ~~(74)~~.

**L647: Insert full stop after sentence.**

**Reply***:* We apologize for the mistake and thank the Reviewer for kindly pointing out the appropriate correction. We have made the necessary adjustments.

**L651: What is meant by “carryover effects” in this context?**

**Reply***: For crossover studies,* “If the effect of a treatment continues after the treatment is withdrawn then the response to a second treatment may well be due in part to the previous treatment” (according to PMID: 8227475). In the case of a study that compares different angles on the same day, a carryover effect could be potentiation or fatigue. We have adjusted the text, as follows (Lines 759-764): An assessment combining both the PEDro and RoB-CO frameworks reveals several concerns, particularly the absence of a clear delineation of methods to eliminate acute carryover effects, such as potentiation or contraction fatigue (40), since some studies compared joint angles on the same day, without justifying the allowed time for recovery between joint angle tests (see “*Bias arising from period and carryover effects*” in Supplemental Digital Content 3).

**L657-659: “…studies have not incorporated a preliminary familiarization session, despite its potential to impact participants' performance”. Insert full stop after sentence. Also, could add doi: 10.1016/j.apmr.2022.09.004. to substantiate this statement.**

**Reply**: We apologize for the mistake and thank the Reviewer for kindly pointing out the appropriate correction. We have added the full stop. Thank you also for pointing out a solid reference to our statement. We have added the new reference (Line 793).

**L663-664: “Quadriceps strength gains are superior at the ideal muscle length, aiding muscle recovery.“ This statement is quite strong from the limited evidence from training studies. Please make it more moderate as there is a scarcity of studies and actual angles tested for training outcomes.**

**Reply**: The insight provided by the reviewer was very helpful. After making robust improvements in the second paragraph of the discussion and other sections, we have added the following statement to the conclusion (Lines 797-802): Quadriceps strength gains ~~are~~ may be superior at the ideal muscle length compared to short muscle lengths, aiding muscle recovery. However, considering the limited NMES evidence, and conflicting findings after voluntary training, the use of short or long muscle lengths should be carefully selected based on diverse clinical factors, such as the available joint range of motion and the need for improvement in muscle-tendon unit properties.

**L723-724: Make “TRANSLATIONAL SPORTS MEDICINE” lower case**

**Reply**: Thank you for noticing this. We have made the necessary correction.

**L843-846: Make ref. 55 lower case. Table 1: The Y/N denotation of PEDro scale evaluation is not easily read. Could a color code be useful here? E.g. white for Y, grey for N. Just a suggestion. Where is the table legend?**

**Reply**: Thank you for noticing this. We have made the necessary adjustments.

**Table 1: Both the PEDro table and the following study characteristics table are called “Table 1”. Please rename. Also, for the characterization table:**

- In the electrode placement of Miyamoto & Oda, 2005: Replace “biceps” with “biceps”

- In the study by Hansen et al. 2003, replace “braquialis” with “brachialis”

- In the study by Mela et al., 2001, replace “Cachode” with “Cathode”

- In the study by Sacco et al.,1994: Replaces “TIbialis” with “Tibialis”

**Reply**: Thank you for noticing and pointing out these mistakes. We have adjusted each one and marked them in red.

**Table 2: In the studies by Cavalcante et al., 2022: The abbreviation “EVA” has not been defined anywhere.
Reply**: Thank you for noticing this. We have made the adjustment.

**Fig 1. from the last step before the screen steps, there are listed ”From database search: n = 826” and ”From other sources: n = 6”, so a total of 832 articles. But in the next step, there are only 826 titles screened. Are the 6 articles from other sources the same as in the last step (”included”)? Also, for the 826 titles screened, 761 records are excluded, giving 65 titles left. The figure shows only 63 titles left. Please check your flow chart for errors in the number of excluded and included articles for each step.**

**Reply:** We apologize to the Reviewer for the mistake and because of the way we constructed the Flowchart, the interpretation may have been misleading (as previously explained in the Results section). We have adjusted Figure 1 (flowchart) to add clarity and have corrected the values. Moreover, the text in the Results section has been adjusted to comply with the new Flowchart (Lines 302-318).

**REVEWER 2**

Dear Reviewer, thank you for your valuable comments. We have tried to address them to the best of our capabilities, and we believe that the manuscript has significantly improved based on your suggestions. Your comments have been addressed point-by-point and are provided below.

**Reviewer #2: Please provide a list of better key words related to the topic of your manuscript. MeSH terms are recommended.**

**Please list reasons for excluding 6 studies that "reports assessed for eligibility in the PRISMA figure. Make sure the numbers in each box add up for example after excluding 761 records you may had 65 studies not 63 as indicated in PRISMA.**

**Please correct the typo on last line of page 7. ...when the data where not available,.... Discussion: The authors have effectively crafted this section, captivating the reader with a wealth of knowledge. To enhance the informative value, it would be beneficial for the authors to explore the impacts of anatomical muscle-tendon-length characteristics, such as the various tendons length of Plantar Flexors to their contractile bulk. This comparison could shed light on the ideal joint angles reported for maximum force production across the examined protocols.**

Reply: Thank you for your valuable comments. We agree with all the recommendations and have adjusted the text accordingly:

- New keywords with MeSH terms have been provided (Lines 79-80);
- A revised and corrected PRISMA figure has been provided; We apologize to the Reviewer for the mistake and because of the way we constructed the Flowchart its interpretation could have been misleading. We have adjusted Figure 1 (flowchart) with clearer information and corrected the values. Moreover, the text in the Results section has been adjusted to comply with the new Flowchart (Lines 302-318);
- The typos have been corrected throughout the manuscript;
- An improved discussion is provided according to the recommendations of both Reviewers, mainly in the following lines where we have included some aspects of tendon loading: 519-523, 550-553, 664-667, 727-730, 798-802.
