# Supplementary material for: Effect of muscle length on maximum evoked torque, discomfort, contraction fatigue, and strength adaptations during electrical stimulation in adult populations: A systematic review
Source: PLoS One. 2024 Jun 10;19(6):e0304205. doi: 10.1371/journal.pone.0304205 (PMC11164398; doi:10.1371/journal.pone.0304205)
Supplement: Attachment — Submitted filename: Comments_2nd round.docx [file pone.0304205.s006.docx]

Line numbers of clean document (without tracked changes):

l. 87:	“As such, the optimal muscle length (or ideal, or preferable) for NMES,..” Parenthesis containing synonyms shouldn’t be in the sentence.

l. 689-692	“… and muscle length comparisons may be influenced by stimulation technique.”

Thank you for this correction. Please add a reference to this statement, e.g. PMID: 12450066
