# Supplementary material for: Effect of muscle length on maximum evoked torque, discomfort, contraction fatigue, and strength adaptations during electrical stimulation in adult populations: A systematic review
Source: PLoS One. 2024 Jun 10;19(6):e0304205. doi: 10.1371/journal.pone.0304205 (PMC11164398; doi:10.1371/journal.pone.0304205)
Supplement: Attachment — Submitted filename: Response to Reviewers.docx [file pone.0304205.s007.docx]

_____________________________________________________________________________________

Jonathan Galvão Tenório Cavalcante MSc,

University of Brasília, Faculdade de Ceilândia

Brasília, DF, Brazil

Centro Metropolitano, Conjunto A, Lote 01

CEP: 70.840-901

Brasília, DF, Brazil

e-mail: jonathangalvao1989@gmail.com

Phone: +55 61 995128423

Fax: +55 61 31078401

May 2, 2024

Dear Professor Dr Theodoros M. Bampouras

Academic Editor, PLOS ONE

Manuscript ID PONE-D-23-35590R1: Effect of muscle length on maximum evoked torque, discomfort, contraction fatigue, and strength adaptations during electrical stimulation in adult populations: a systematic review

Thank you for the opportunity to answer the reviewer and upload our revised manuscript.

We are glad that the reviewer is happy with the amendments we made based on the suggestions we received. We now hapilly receive the relevant minor corrections pointed out. A point-by-point response to the reviewer is provided bellow, and the marked-up, and unmarked versions of the manuscript were uploaded.

Thank you again for your time and effort in considering this manuscript for publication.

Sincerely,

Jonathan Galvão Tenório Cavalcante

João Luiz Quagliot Durigan

Laboratory of Muscle and Tendon Plasticity

Graduate Program in Rehabilitation Sciences

University of Brasília,

**TO THE ACADEMIC EDITOR**

***“If you would like to make changes to your financial disclosure, please include your updated statement in your cover letter. Guidelines for resubmitting your figure files are available below the reviewer comments at the end of this letter.”***

**Reply:** Thank you for the information. No change is necessary.

***“If applicable, we recommend that you deposit your laboratory protocols in protocols.io to enhance the reproducibility of your results. Protocols.io assigns your protocol its own identifier (DOI) so that it can be cited independently in the future. For instructions see: https://journals.plos.org/plosone/s/submission-guidelines#loc-laboratory-protocols. Additionally, PLOS ONE offers an option for publishing peer-reviewed Lab Protocol articles, which describe protocols hosted on protocols.io. Read more information on sharing protocols at https://plos.org/protocols?utm_medium=editorial- email&utm_source=authorletters&utm_ campaign=protocols.”***

**Reply:** Thank you. Not applicable.

***“Please review your reference list to ensure that it is complete and correct. If you have cited papers that have been retracted, please include the rationale for doing so in the manuscript text, or remove these references and replace them with relevant current references. Any changes to the reference list should be mentioned in the rebuttal letter that accompanies your revised manuscript. If you need to cite a retracted article, indicate the article’s retracted status in the References list and also include a citation and full reference for the retraction notice.”***

**Reply:** Thank you for pointing to this relevant double check in our reference list. We did not cite retracted papers. We added a new reference based on reviewer’s suggestion: “*87.	Rassier DE, MacIntosh BR. Length-dependent twitch contractile characteristics of skeletal muscle. Can J Physiol Pharmacol. 2002;80(10):993-1000.”*

***“While revising your submission, please upload your figure files to the Preflight Analysis and Conversion Engine (PACE) digital diagnostic tool, https://pacev2.apexcovantage.com/. PACE helps ensure that figures meet PLOS requirements. To use PACE, you must first register as a user. Registration is free. Then, login and navigate to the UPLOAD tab, where you will find detailed instructions on how to use the tool. If you encounter any issues or have any questions when using PACE, please email PLOS at figures@plos.org. Please note that Supporting Information files do not need this step.”***

**Reply:** Thank you for the helpful tool. The last upload of our figures (during the first review) was performed after using PACE to make the files meet PLOS requirements.

**REVIEWER 1**

Dear Reviewer, thank you again for your valuable comments and for appreciating our first rebuttal letter. We are confident that the manuscript has significantly improved based on your suggestions. Your comments have been addressed point-by-point and are provided below.

**Reviewer #1: *“I thank the authors for their corrections and further considerations on their manuscript. I have only a few minor comments to the current manuscript:***

***Line numbers of clean document (without tracked changes):***

***l. 87: “As such, the optimal muscle length (or ideal, or preferable) for NMES,..”***

***It would be preferable if you could settle on one of the words and delete the parenthesis.***

**Answer:** Thank you for mentioning this necessary adjustment. We have adjusted the text accordingly: “*As such, the optimal muscle length ~~(or ideal, or preferable)~~ for NMES…”*

***l. 689-692 “… and muscle length comparisons may be influenced by stimulation technique.”***

***Thank you for this correction. Please add a reference to this statement, e.g. PMID: 12450066***
**Answer:** Thank you for the suggestion. We agree that a sound reference was missing and we considered appropriate to add the suggested reference: “*and muscle length comparisons may be influenced by stimulation technique (87).”*

87.	Rassier DE, MacIntosh BR. Length-dependent twitch contractile characteristics of skeletal muscle. Can J Physiol Pharmacol. 2002;80(10):993-1000.
